# *Easi-*CRISPR: a robust method for one-step generation of mice carrying conditional and insertion alleles using long ssDNA donors and CRISPR ribonucleoproteins

**DOI:** 10.1186/s13059-017-1220-4

**Published:** 2017-05-17

**Authors:** Rolen M. Quadros, Hiromi Miura, Donald W. Harms, Hisako Akatsuka, Takehito Sato, Tomomi Aida, Ronald Redder, Guy P. Richardson, Yutaka Inagaki, Daisuke Sakai, Shannon M. Buckley, Parthasarathy Seshacharyulu, Surinder K. Batra, Mark A. Behlke, Sarah A. Zeiner, Ashley M. Jacobi, Yayoi Izu, Wallace B. Thoreson, Lisa D. Urness, Suzanne L. Mansour, Masato Ohtsuka, Channabasavaiah B. Gurumurthy

**Affiliations:** 1 Mouse Genome Engineering Core Facility, Vice Chancellor for Research Office, University of Nebraska Medical Center, Omaha, NE USA; 2 Department of Molecular Life Science, Division of Basic Medical Science and Molecular Medicine, Tokai University School of Medicine, 143 Shimokasuya, Isehara, Kanagawa, 259-1193 Japan; 3 Center for Matrix Biology and Medicine, Graduate School of Medicine, Tokai University, 143 Shimokasuya, Isehara, Kanagawa 259-1193 Japan; 4 Department of Host Defense Mechanism, Division of Basic Medical Science and Molecular Medicine, Tokai University School of Medicine, 143 Shimokasuya, Isehara, Kanagawa 259-1193 Japan; 5 Laboratory of Molecular Neuroscience, Medical Research Institute (MRI), Tokyo Medical and Dental University (TMDU), 1-5-45, Yushima, Bunkyo, Tokyo, 113-8510 Japan; 6 Laboratory of Recombinant Animals, MRI, TMDU, 2-3-10, 2-3-10, Surugadai, Kanda, Chiyoda, Tokyo, 101-0062 Japan; 7 Present address: McGovern Institute for Brain Research, Massachusetts Institute of Technology, Cambridge, MA 02139 USA; 8 High-Throughput DNA Sequencing and Genotyping Core Facility, Vice Chancellor for Research Office, University of Nebraska Medical Center, Omaha, NE USA; 9 Sussex Neuroscience, University of Sussex, Falmer, Brighton, BN1 9QG UK; 10 The Institute of Medical Sciences, Tokai University, 143 Shimokasuya, Isehara, Kanagawa 259-1193 Japan; 11 Department of Regenerative Medicine, Tokai University School of Medicine, 143 Shimokasuya, Isehara, Kanagawa 259-1193 Japan; 12 Department of Orthopaedic Surgery, Tokai University School of Medicine, 143 Shimokasuya, Isehara, Kanagawa 259-1193 Japan; 13 Department of Genetics, Cell Biology & Anatomy, College of Medicine, University of Nebraska Medical Center, Omaha, NE USA; 14 Department of Biochemistry and Molecular Biology, University of Nebraska Medical Center, Omaha, NE USA; 15 Fred and Pamela Buffett Cancer Center, Eppley Institute for Research in Cancer and Allied Disease, University of Nebraska Medical Center, Omaha, NE USA; 16 Integrated DNA Technologies, Inc., Coralville, IA 52241 USA; 17 Department of Animal Risk Management, Chiba Institute of Science, 3 Shiomi-cho, Choshi, Chiba 288-0025 Japan; 18 Truhlsen Eye Institute and Department of Ophthalmology & Visual Sciences, University of Nebraska Medical Center, Omaha, NE 68198 USA; 19 Department of Human Genetics, University of Utah, Salt Lake City, UT 84112 USA; 20 Developmental Neuroscience, Munroe Meyer Institute for Genetics and Rehabilitation, University of Nebraska Medical Center, Omaha, NE USA

**Keywords:** CRISPR/Cas9, Homology directed repair, *Easi-*CRISPR, long ssDNA donors, CRISPR ribonucleoproteins, Cre-LoxP, Conditional knockout, Reporter and recombinase knock-in

## Abstract

**Background:**

Conditional knockout mice and transgenic mice expressing recombinases, reporters, and inducible transcriptional activators are key for many genetic studies and comprise over 90% of mouse models created. Conditional knockout mice are generated using labor-intensive methods of homologous recombination in embryonic stem cells and are available for only ~25% of all mouse genes. Transgenic mice generated by random genomic insertion approaches pose problems of unreliable expression, and thus there is a need for targeted-insertion models. Although CRISPR-based strategies were reported to create conditional and targeted-insertion alleles via one-step delivery of targeting components directly to zygotes, these strategies are quite inefficient.

**Results:**

Here we describe *Easi-*CRISPR (*E*fficient *a*dditions with *s*sDNA *i*nserts-CRISPR), a targeting strategy in which long single-stranded DNA donors are injected with pre-assembled crRNA + tracrRNA + Cas9 ribonucleoprotein (ctRNP) complexes into mouse zygotes. We show for over a dozen loci that *Easi*-CRISPR generates correctly targeted conditional and insertion alleles in 8.5–100% of the resulting live offspring.

**Conclusions:**

*Easi-*CRISPR solves the major problem of animal genome engineering, namely the inefficiency of targeted DNA cassette insertion. The approach is robust, succeeding for all tested loci. It is versatile, generating both conditional and targeted insertion alleles. Finally, it is highly efficient, as treating an average of only 50 zygotes is sufficient to produce a correctly targeted allele in up to 100% of live offspring. Thus, *Easi-*CRISPR offers a comprehensive means of building large-scale Cre-*LoxP* animal resources.

**Electronic supplementary material:**

The online version of this article (doi:10.1186/s13059-017-1220-4) contains supplementary material, which is available to authorized users.

## Background

Conditional knockout mouse models, in which one or more critical coding exons of a gene are flanked by similarly oriented *LoxP* sites (i.e., floxed), are among the most useful genetically engineered models in biomedical research. They provide the opportunity to define essential gene functions in both global and tissue-specific contexts [1, 2] and are particularly critical for analyzing genes that have essential functions early in development. Indeed, several large-scale global projects pursued under the umbrella of the International Mouse Phenotyping Consortium (IMPC) set a collective goal of generating a floxed or deletion allele for each mouse gene and to make these alleles readily available to the research community [3, 4]. To date, this goal has been pursued using traditional strategies that rely on homologous recombination (HR) in embryonic stem (ES) cells to deliver targeting cassettes flanked by long regions of homology (~3–10 kb) to the gene of interest [5], followed by appropriate selection techniques. Correctly targeted ES cells are then introduced into mouse embryos, and the resulting chimeric mice are used to transfer the floxed allele to subsequent generations. The time required to generate floxed mice by the standard method is at least 6 months, even when starting with an ES cell line procured from one of the repositories. Furthermore, only about 25% of mouse genes have been targeted in this way, and the genetic background of ES cells used by the consortium is limited to the C57BL/6 strain, which, though a reference strain, is not ideal for all purposes [6, 7].

CRISPR/Cas9-directed genome editing should, in theory, allow for the more rapid generation of floxed alleles in any chosen genetic background, because the editing components can be delivered directly to single-cell mouse zygotes of any strain. Indeed, within months of the first demonstration of CRISPR/Cas9 genome editing to produce small gene disruptions in mammalian cells [8, 9], a proof-of-concept study showed that conditional knockout mice could be generated by homology-directed repair (HDR) following injection of mouse zygotes with five components: two separate single guide RNAs (sgRNAs) targeted to sequences flanking an exon of interest; two single-stranded oligodeoxynucleotide (ssODN) donors, each containing a *LoxP* site flanked by short (40–80 bases) arms homologous to the desired insertion site; and *Cas9* mRNA. Using this method, the authors found that *Mecp2* was correctly floxed in 16% of the embryos/mice derived after transfer of injected zygotes [10]. To date, however, only two other reports have been published showing that floxed mice can be generated using this approach. Bishop et al. [11] and Miano et al. [12] reported efficiencies of *LoxP* integration of 2–5% and identified some of the reasons for its poor success. A recent news article in *Science* reported anecdotal evidence that this method has been unsuccessful at many loci, and that cases of successful CRISPR-directed floxing had efficiencies of only 1 or 2% [13]. A major factor limiting the targeting efficiency of this approach is the complex set of modifications that the targeting components can generate in addition to the desired insertion of two *LoxP* sites located *in cis*. These include single *LoxP* insertions, double *LoxP* insertions located *in trans*, and a variety of deletions resulting from non-homologous end-joining (NHEJ), all of which may vary in a locus-dependent manner [10, 11]. Thus, this appealingly simple and rapid two-ssODN donor approach is not robust enough for routine generation of floxed alleles.

As an alternative to short ssODN donors, insertions of longer sequences (floxed exons or coding sequences) have been attempted using double-stranded DNA (dsDNA) donors with homology arms of at least 0.5–1 kb. Compared with ssODN donors, the insertion efficiency of dsDNA donors is often poor [10, 14–16]. For example, an IMPC study showed that classic HR-mediated cassette insertion could be achieved directly in zygotes by creating two nicks near the target site using Cas9 nickase and co-injecting a floxed donor cassette of dsDNA with homology arms of ~1.9 kb [17]. However, only one out of thirteen pups born contained the desired allele, and this approach has not been used routinely.

Other strategies for increasing the efficiency of CRISPR/Cas9 genome editing include inhibition of NHEJ or enhancement of HDR through chemical treatments [18, 19]. These approaches, however, are based on perturbation of DNA repair processes and may be toxic [20]. Additional strategies include the use of circular donors with built-in artificial guide sequences that are linearized inside the cell/embryo, wherein donors are inserted at the genomic Cas9 cleavage site by cellular ligases [21–23]. These targeting designs include either micro-homology ends between the cleaved ends of the genomic DNA and donor DNA, or ssODNs that bind to the two free ends so that precise fusion occurs between the donor and genomic DNAs. Although these latter strategies offer better alternatives to those that perturb DNA repair, they too have limitations, including low-to-moderate efficiencies and the need for custom design of donor plasmids for each target site. Due to the poor efficiency of direct zygote injections, some groups have also tried to develop CRISPR/Cas9-based strategies for creating knock-ins via ES cell targeting [20, 24, 25]. Although these proved feasible, they are neither efficient nor robust enough for routine application.

Because short ssODN donors are inserted efficiently at Cas9 cleavage sites through an HDR pathway, we reasoned that this repair mechanism might be exploited to deliver longer cargo if the length of the single-stranded DNA (ssDNA) could be extended. Based on our experience with using ssDNA donors and an sgRNA to insert ~400-base fragments into the mouse genome with high efficiency when assayed at embryonic stages [26], we asked whether longer ssDNA donors and two guide RNAs could be used to generate mice with floxed exons. Here, we demonstrate that long ssDNA donors with short homology arms generate conditional knockout mice at high efficiency when using pre-assembled crRNA + tracrRNA + Cas9 ribonucleoprotein (ctRNP) complexes containing two guide RNAs. We also show that knock-ins of reporter, recombinase, and transcriptional effector genes can be generated at similar efficiencies, by providing long ssDNA donors with ctRNPs that contain one guide RNA. Our method, called *Easi*-CRISPR (*E*fficient *a*dditions with *s*sDNA *i*nserts-CRISPR), is robust and, having been tested at more than a dozen loci (creating seven floxed and six knock-in alleles), is also highly generalizable. *Easi*-CRISPR thus provides a comprehensive solution to the challenges of generating both necessary components (floxed and Cre alleles) for conditional gene ablation in mice, as well as enabling rapid development of numerous other desired alleles.

## Results

### Efficient generation of floxed alleles using long ssDNA donors

As a test case, we selected *Pitx1* and generated a 1046-base ssDNA donor containing a floxed version of exon 2, flanked by 93- and 91-base left and right homology arms, respectively. Two guide RNAs (sgRNAs) were designed to cut the genome immediately adjacent to each homology arm (Additional file 1: Figure S1a). We injected the ssDNA donor with *Cas9* mRNA and the two sgRNAs into mouse zygotes following standard CRISPR genome engineering protocols [27]. Genotyping of the resulting live offspring, using three sets of PCR reactions specific for targeted insertion of each *LoxP* site and for the entire floxed exon, revealed that one out of eight (13%) carried a correctly floxed allele (Additional file 1: Figure S1b–g). Three other pups had partial insertions of the donor cassette: two contained only a single targeted *LoxP* site and one contained both *LoxP* insertions, but they were located on separate alleles (*in trans*).

**Fig. 1:**
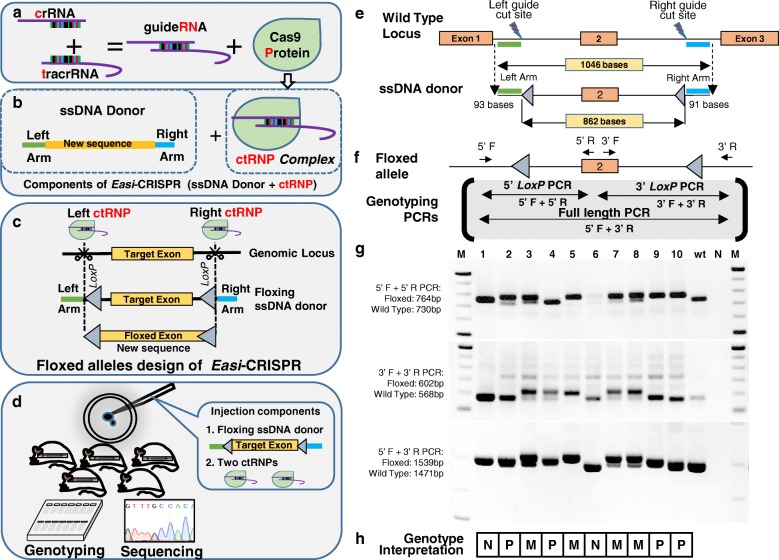
Generation of a floxed *Pitx1* allele using *Easi*-CRISPR. **a**–**d** The *Easi-*CRISPR strategy. **a** The two parts of the CRISPR guideRNA (crRNA + tracrRNA) and Cas9 protein. Combining them generates a ctRNP complex. The term ctRNP used here was formerly known as a cloning-free CRISPR/Cas9 system [28]. **b** A long ssDNA donor derived from a floxed exon cassette (or knock-in cassette as in Fig. 3) is mixed with ctRNP(s) to obtain the final *Easi-*CRISPR reagent cocktail for zygote injection. **c** Injection of a floxed ssDNA donor with right and left ctRNPs into zygotes results in replacement of the target exon with the floxed exon. For targeted insertions (as in Fig. 3) only a single ctRNP is required. **d** Following microinjection of the *Easi*-CRISPR reagent cocktail, genotyping and sequencing are used to identify founders with correctly modified genomes. **e**, **f** The *Pitx1* wild-type allele, the *Pitx1* ssDNA donor designed to flox exon 2 and the final targeted allele. The lengths of ssDNA, homology arms, and the distance between the two *LoxP* sites are shown. **f** Three genotyping PCRs and the primer combinations for these are indicated (5′ *LoxP* PCR, 5′ F + 5′ R primers; 3′ *LoxP* PCR, 3′ F + 3′ R primers; and full-length PCR, 5′ F + 3′ R primers). **g** Genotyping gel images from the ears of G0 offspring. The expected sizes of PCR amplicons (wild type (*wt*) or floxed) are indicated to the left of the gels. **h** Genotype interpretations are summarized below the gel image (*M* monoallelic, *P* partial insertion, *N* no insertion). Animals 3, 5, 7, and 8 had both the 5′ and 3′ *LoxP* sites in *cis*, while animals 2, 4, 9, and 10 contained only one *LoxP* site, due to partial insertion of the ssDNA cassette

We suspected that the partial insertions might be a result of using *Cas9* mRNA, which must first be translated to produce Cas9 protein, and that low protein levels might reduce the probability of simultaneously cleaving both sites on the same allele. It was demonstrated previously that ssODN donors promote increased frequencies of HDR when they are delivered with a ribonucleoprotein (RNP) complex comprised of Cas9 protein and separated guide RNAs (crRNA + tracrRNA) relative to when they are delivered with Cas9 in complex with sgRNA or with a mix of *Cas9* mRNA with sgRNA [28]. To determine whether a similar approach could enhance the frequency of HDR with long ssDNA donors, we prepared a crRNA + tracrRNA + Cas9 protein complex using chemically synthesized crRNAs and tracrRNAs designed to cleave *Pitx1* in exactly the same sites as the sgRNAs described above. Hereafter we refer to crRNA + tracrRNA + Cas9 complexes as ctRNPs to avoid confusion with sgRNA/Cas9 RNP complexes, which are called sgRNPs. The ctRNP complex was mixed with the same *Pitx1* floxed exon 2 ssDNA donor used previously and injected into zygotes. Genomic DNA from the resulting offspring were genotyped and those with *Pitx1* insertions were sequenced. Schematics of the workflow of *Easi*-CRISPR with ctRNPs and the details of the *Pitx1* donor design and genotyping PCRs are shown in Fig. 1a–f. We obtained ten live offspring from these *Easi*-CRISPR with ctRNP injections. Two animals had no insertions, four had partial insertions of a single *LoxP* site, and four had bona fide floxed alleles (40% correct insertion; Fig. 1g, h).

**Fig. 2:**
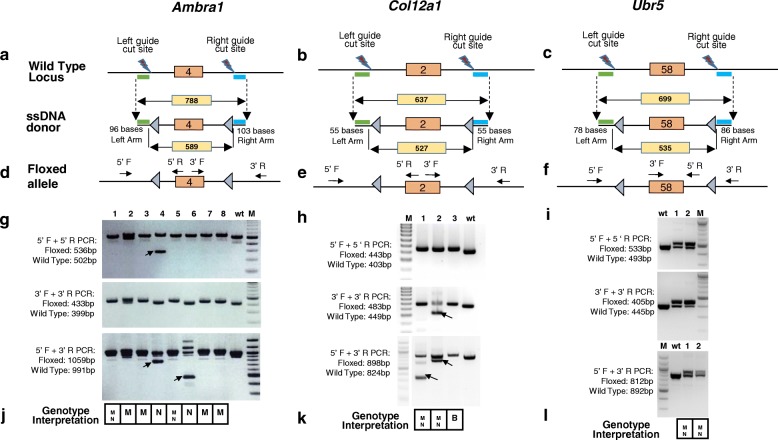
Generation of floxed alleles for *Ambra1*, *Col12a1*, and *Ubr5* using *Easi*-CRISPR. **a**–**c** The wild-type alleles, floxing ssDNA donors for targeting exons 4, 2, and 58 of *Ambra1*, *Col12a1*, and *Ubr5*, respectively, and the corresponding floxed alleles. The lengths of ssDNA, homology arms, and the distance between the two *LoxP* sites are shown. **d**–**f** The primer pairs and genotyping PCRs are indicated as in Fig. 1f. The floxed allele schematics show minor differences in primer locations for each gene with respect to target exon size and location. **g**–**i** Genotyping of G0 offspring. The expected sizes of PCR amplicons (wild type or floxed) are indicated to the left of the gels. **j**–**l** Genotype interpretations are summarized below the gel images (*M* monoallelic, *B* biallelic, *N* no insertion). **j** Interpretation of *Ambra1* genotyping: animals 1, 2, 3, 5, 7, and 8 had both the 5′ and 3′ *LoxP* sites located in *cis*. Note that animals 4 and 6 also contain additional amplicons smaller than the expected size (shown by *arrows*), suggesting that they harbor deletions and/or are mosaic. The sequences of the deletion alleles were not determined. Animals 1 and 5 were bred to wild type and a CD4 Cre mouse line (Fig. 5; Additional file 1: Figure S4). **k** Interpretation of *Col12a1* genotyping: animals 1 and 2 were heterozygous for both 5′ and 3′ *LoxP* sites located in *cis*, and they carried deletions in their second allele (shown by the *arrows*); animal 3 was biallelic for both the 5′ and 3′ *LoxP* sites. The lanes between the marker and the samples in the full-length PCR gel image (*bottom panel*) were cropped out because they belonged to another experiment. **l** Interpretation of *Ubr5* genotyping: animals 1 and 2 were heterozygous for both 5′ and 3′ *LoxP* sites located in *cis*

Encouraged by this result, we asked whether similarly high targeting efficiencies could be obtained at other loci. We selected six more genes (*Ambra1*, *Col12a1*, *Ubr5*, *Syt1*, *Syt9*, and *Ppp2r2a*) to generate floxed alleles using *Easi*-CRISPR with ctRNPs. Details of the target exons, the lengths of the ssDNA repair templates, homology arms, and genotyping strategies are shown in Fig. 2 and Additional file 1: Figure S2. The microinjection details and the efficiencies of precise floxing are shown in Table 1. Our targeting strategy succeeded for all six genes, with efficiencies ranging from 8.5 to 100%. Of note, at least two founder pups contained biallelic insertions of the donor cassettes (Fig. 2h, *Col12a1* ^*flox*^ #3; Additional file 1: Figure S2i, *Ppp2r2a* ^*flox*^ #3; Table 1).

**Table 1:** Microinjection data for floxed allele generation at seven loci

*Gene*-insertion cassette	ssDNA length Left Arm-Cassette-Right Arm (bases) [source of ssDNA]	Zygotes injected	Zygotes transferred	Live-born animals (percentage of transferred zygotes)	Targeted animals (%)^a^
*Pitx1*-exon 2 floxed	93 + 862 + 91 [*Iv*TRT]	85	76	10 (13.2)	4 (40%)^b^
*Ambra1*-exon 4 floxed	96 + 589 + 103 [*Iv*TRT]	67	63	8 (12.7)	6 (75%)^c^
*Col12a1-*exon 2 floxed	55 + 527 + 55 [*Iv*TRT]	105	79	3 (3.8)	3 (100%)^d^
*Ubr5*-exon 58 floxed	78 + 535 + 86 [*Iv*TRT]	20	16	2 (12.1)	2 (100%)^e^
*Syt1*-exon 6 floxed	75 + 635 + 75 [IDT Megamer™]	51	45	8 (17.8)	1 (12.5%)^f^
*Syt9*-exon 3 floxed	87 + 893 + 68 [IDT Megamer™]	43	41	12 (29.3)	1 (8.5%)^g^
*PPP2r2a*-exon 3 floxed	95 + 619 + 84 [IDT Megamer™]	34	33	3 (9.1)	3 (100%)^h^

^a^The alleles that did not contain the inserts were not analyzed for the presence of *indels* because genotyping assays were mainly designed to identify the targeted-insertion alleles. However, noticeable deletions were observed for some samples (e.g., deletions in the non-targeted alleles; Fig. 2g, h; Additional file 1: Figure S2i)

^b^Animals 3, 5, 7, and 8 were heterozygous for both 5′ and 3′ *LoxP* sites. Animal 5 had a floxed allele with one nucleotide insertion mutation at the intronic region, which may not affect function. Animals 2, 9, and 10 had only 5′ *LoxP* site, and animal 4 had only 3′ *LoxP* site (Fig. 1g)

^c^Animals 1, 2, 3, 5, 7, and 8 were heterozygous for both the 5′ and 3′ *LoxP* sites (Fig. 2g). Animal 7 had a floxed allele with 1-bp insertion mutation in the intronic region, which may not affect function

^d^Animals 1 and 2 were heterozygous for both 5′ and 3′ *LoxP* sites and they carried deletions in their second allele. Animal 3 was biallelic for both *LoxP* sites (Fig. 2h)

^e^Animals 1 and 2 were heterozygous for both 5′ and 3′ *LoxP* sites (Fig. 2i)

^f^Animals 4 and 7 had only 5′ *LoxP* insertion and the animal 6 had correctly targeted *LoxP* sites (Additional file 1: Figure S2g)

^g^Animal 12 had correctly targeted *LoxP* sites and all others were wild type (Additional file 1: Figure S2h).

^h^Animals 1 and 2 were heterozygous for both *loxP*s with deletions in the second allele and pup 3 was biallelic (Additional file 1: Figure S2i)

To directly compare *Easi*-CRISPR with the previously described method for generating floxed alleles [10], we targeted the same *Pitx1* exon using two guides and two short ssODN donors containing the *LoxP* sites (Additional file 1: Figure S3a). We prepared the *Pitx1* ctRNP exactly as described above and injected it, together with the two ssODN donors, into 66 zygotes, from which 18 animals were born. Genotyping showed that many animals carried a single *LoxP* site (three had only the 5′ *LoxP* and three had only the 3′ *LoxP*). Only one of the 18 animals contained both *LoxP* sites on the same allele (*in cis*; Additional file 1: Figures S3b, c, lane 2). However, the sequence of the distal *LoxP* site contained a mutation (Additional file 1: Figure S3d), and therefore this animal would not be useful for conditional deletion of *Pitx1*; similar unwanted mutations, in *LoxP* sites, were reported previously for another locus [12]. Of note, even the genomes that had single *LoxP* insertions also contained various types of deletions (evident by differently sized PCR products; Additional file 1: Figure S3c, lanes 5, 8, 14, and16). These results clearly confirm that although various types of insertion events can occur when using the two-ssODN donor method, it is quite challenging to identify and/or obtain correctly targeted animals. These observations are similar to those made by others [11, 12].

In summary, for all seven genes combined, genotyping of 46 G0 pups showed that 20 (43%) contained at least one correctly floxed allele, with an efficiency ranging from 8.5–100% at different loci. The fidelity of the insertions and correct fusions was confirmed by sequencing (Additional file 1: Figures S4–S10). Of the 20 founders with correctly floxed exons, two contained point mutations in the inserted regions (one each for *Pitx1* and *Ambra1*) that may have derived from enzymatic misincorporation during preparation of the ssDNA donor templates. Nevertheless, such mutations did not affect the overall goal of generating floxed mice because we obtained at least one founder with a correct insertion for each gene. Moreover, even the founders with mutations are potentially useful because the mutations were located in intronic sites that are less likely to affect gene function.

### Efficient generation of knock-in alleles using long ssDNA donors

**Fig. 3:**
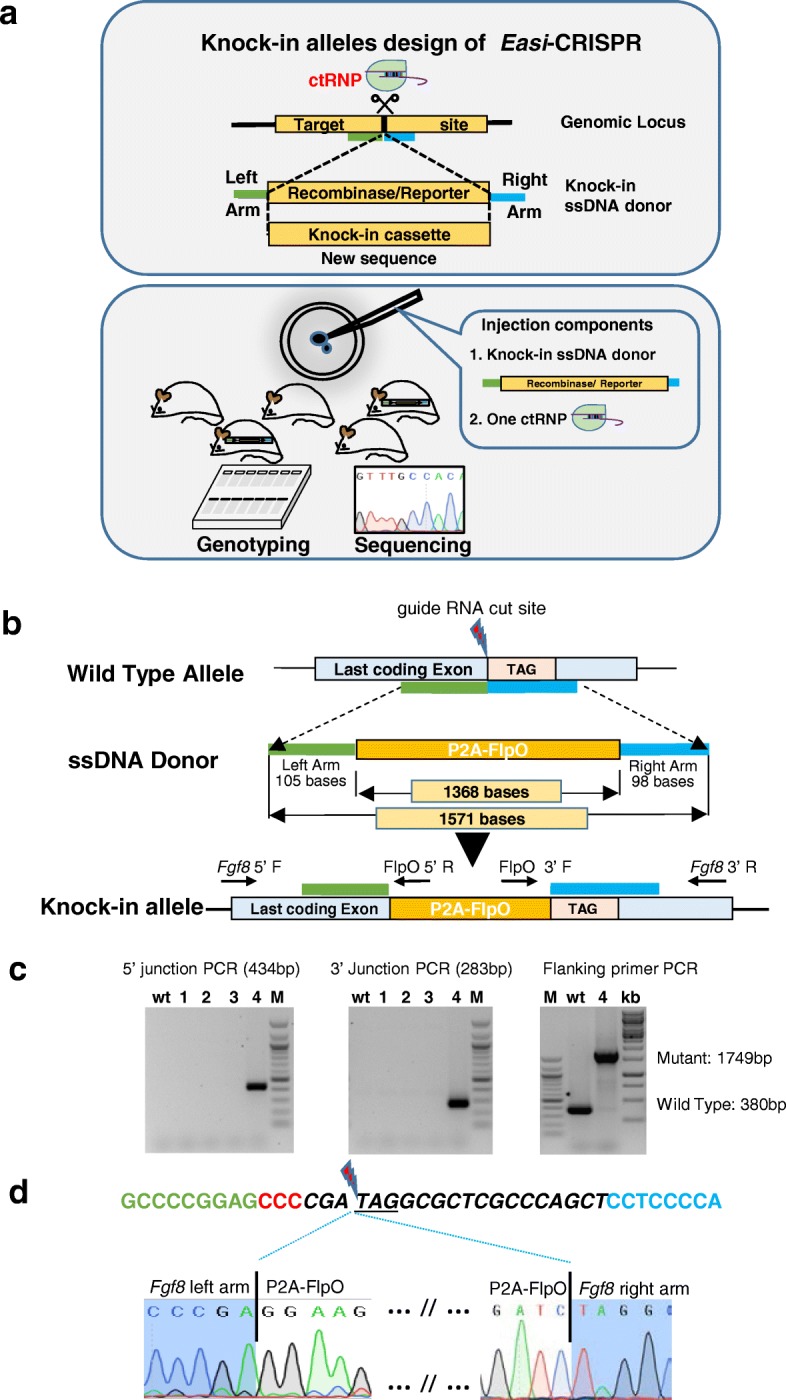
Fusion of P2A-FlpO to the 3′ end of *Fgf8* using *Easi*-CRISPR. **a** How *Easi-*CRISPR is used to generate knock-in alleles. **b** The *Fgf8* locus, ssDNA donor, and the resulting targeted insertion allele. **c** Genotyping of G0 offspring. Primer locations for 5′ and 3′ junction PCRs are shown, along with expected amplicon sizes. Founder 4 has a correctly targeted P2A-FlpO insertion, as indicated by the presence and size of both 5′ and 3′ junction amplicons. The gel on the *right* shows that PCR amplification of this founder’s DNA with primers flanking the *Fgf8* insertion site produced only the mutant amplicon, indicating that it is a biallelic insertion. *WT* wild type, *M* 100-bp marker; *kb* 1-kb marker. **d** Sequencing of 5′ and 3′ junctions in founder 4*.* The guide RNA sequence (*italics*), along with the cut site, PAM sequence (in *red*), a few bases of flanking sequences (*above*) and sequence chromatograms showing correctly targeted 5′ and 3′ junctions are shown below

Based on the success of *Easi*-CRISPR for floxing various loci, we asked whether similar efficiencies could be obtained for knock-ins of sequences that encode reporters, recombinases, and transcriptional regulators. We designed ssDNA donors and the appropriate guide RNAs to target six different loci. The ssDNA donor cassettes consisted of sequences ranging from 0.8–1.4 kb, and encoded either FlpO recombinase, the reverse tetracycline transactivator (rtTA), or the reporters mCherry and mCitrine (Table 2). As with the donors designed for floxing, these inserts were flanked by homology arms of 60–105 bases. Schematics of the ssDNA cassettes, lengths of homology arms, and knock-in cassettes are shown in Fig. 3a, b and Additional file 1: Figures S11a–S15a and their full sequences are shown in Additional file 1: Figures S16–S21. PCR genotyping of offspring indicated that targeted insertion efficiencies for the different genes ranged from 25–67% (Fig. 3c; Additional file 1: Figures S11b–S15b; Table 2). Correct targeting was confirmed by sequencing the expected 5′ and 3′ junction fragments (Fig. 3d; Additional file 1: Figures S11c–S15c). Of the 39 pups analyzed, 17 (44%) had the expected sequence at both junctions. Although three more pups contained targeted insertions, they were not perfect at their 3′ junctions; two of the pups contained extra sequences (e.g., *Slc26a5* ^P2A-FlpO^ #1 (Additional file 1: Figure S11b) and *Mmp9* ^T2A-mCitrine^ #10 (Additional file 1: Figure S14b)), and the third pup lacked some of the donor sequence (e.g., *Mmp13* ^T2A-mCherry^ #2 (Additional file 1: Figure S15b)). Of note, one founder for *Fgf8* ^P2A-FlpO^ contained biallelic insertions of the knock-in cassette (Fig. 3c). The sequences of the inserts were accurate in 12 of the 17 founders. The remaining five animals (one each for *Fgf8*, *Slc26a5*, *Mafb*, *Mmp9*, and *Mmp13* founders) contained point mutations in their knock-in cassettes that may have derived from enzymatic misincorporation during preparation of ssDNA donor templates. *Easi-*CRISPR was repeated for *Fgf8* ^P2A-FlpO^ because only one knock-in founder was initially produced and it contained a non-synonymous mutation in the FlpO cassette. Similarly, only one knock-in *Otoa* ^rtTA^ founder was born and it was a runt that did not survive past 5 weeks of age. The second batch of experiments resulted in two out of six *Fgf8* ^P2A-FlpO^ and three out of eight *Otoa* ^rtTA^ live-born animals carrying the desired knock-in (Additional file 1: Figure S22). The efficiencies of knock-ins were comparable between the two independent sessions of microinjections—25 versus 33% for *Fgf8* ^P2A-FlpO^ and 50 versus 37.5% for *Otoa* ^rtTA^—demonstrating the reproducibility of our method.

**Table 2:** Microinjection data for knock-in allele generation at six loci

*Gene*-insertion cassette	ssDNA length Left Arm-Cassette-Right Arm (bases) [source of ssDNA]	Zygotes injected	Zygotes transferred	Live-born animals (percentage of transferred zygotes)	Targeted animals (%)^a^
*Fgf8*-P2A-FlpO	105 + 1368 + 98 [IDT Megamer™]	22	13	4 (30.8)	1 (25%)^b^
*Slc26a5*-P2A-FlpO	99 + 1368 + 72 [IDT Megamer™]	28	22	3 (13.6)	1 (33%)^c^
*Mafb*-P2A-FlpO	85 + 1368 + 96 [IDT Megamer™]	58	53	8 (15.1)	2 (25%)
*Otoa*-rtTA	96 + 1220 + 98 [IDT Megamer™]	19	18	2 (11.1)	1 (50%)
*Mmp9-*T2A-mCitrine	60 + 782 + 60 [*Iv*TRT]	52	50	12 (24)	8 (67%)^d^
*Mmp13-*T2A-mCherry	60 + 779 + 60 [*Iv*TRT]	55	52	10 (19.2)	4 (40%)

^a^The alleles that did not contain the inserts were not analyzed for the presence of *indels* because genotyping assays were mainly designed to identify the targeted-insertion alleles. However, noticeable sequence additions or deletions were observed for some samples in these assays (e.g., additions in *Slc26a5* animal 1 (Additional file 1: Figure S11), in *Mmp9* animal 10 (Additional file 1: Figure S14), and deletion in *Mmp9* animal 4 (Additional file 1: Figure S14))

^b^Animal 4 had bi-allelic insertions of the knock-in cassette (Fig. 3c)

^c^Animal 1 had additional sequences at the 3′ junction (sequence not fully characterized and pup 3 had a precise insertion at both junctions (Additional file 1: Figure S11)

^d^Animal 4 appeared to be mosaic containing both a correctly targeted allele and a deletion in the 3′ junction (sequence not fully characterized) (Additional file 1: Figure S14)

### *Easi-*CRISPR founders transmit their modified alleles to offspring, which show the expected phenotypes

**Fig. 4:**
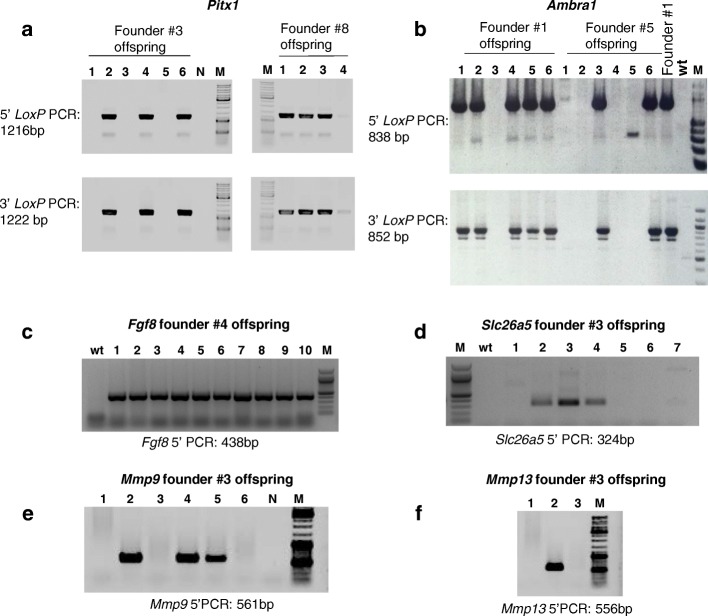
Germ line transmission of founder alleles generated using *Easi-*CRISPR. **a**–**f** Genotyping of offspring from two founders each for the *Pitx1* and *Ambra1* conditional alleles (**a**, **b**) and one founder each of the *Fgf8*, *Slc26a5*, *Mmp9*, and *Mmp13* knock-in alleles (**c**–**f**) showing germ line transmission from all of these founders. As expected, all the pups from the *Fgf8* founder contain a targeted allele because the founder is biallelic (**c**)

**Fig. 5:**
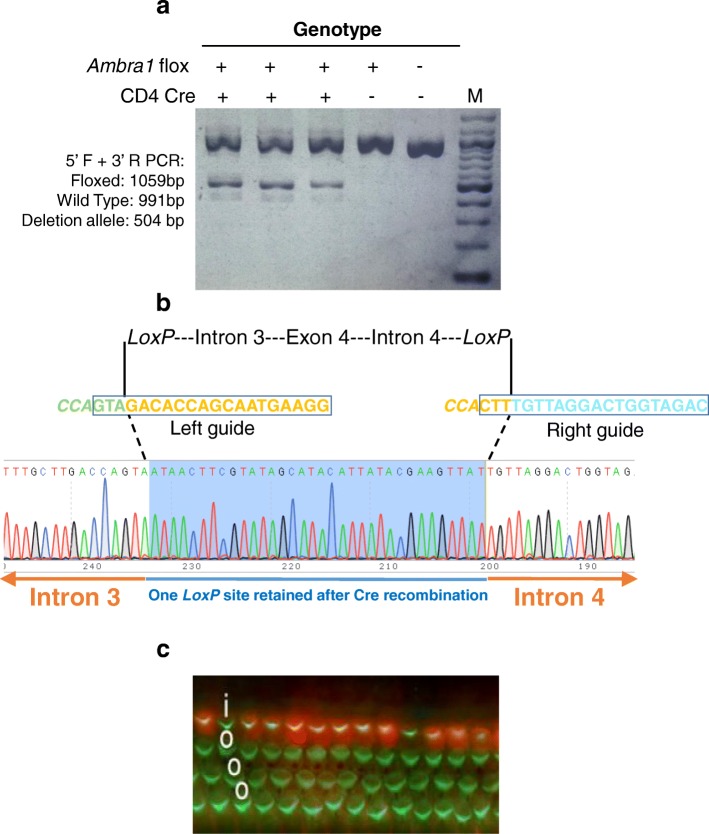
*Easi-*CRISPR alleles perform as intended. Conditional alleles show the expected pattern of Cre-mediated deletion. **a** Genotyping of lymphocyte DNA isolated from a litter produced by mating the *Ambra1* floxed founder 1 (Fig. 2g, lane 1) with a CD4 Cre strain. Offspring carrying both the floxed and Cre alleles (first three lanes) show the expected PCR amplicons. *wt* wild-type control sample, *M* 100-base pair marker. **b** Sequencing of a deletion allele showing Cre recombination (see Additional file 1: Figure S5 for comparing this sequence with the floxed allele sequence). **c** *FGF8*-P2A-FlpO activates a FLP-dependent tdTomato reporter in inner hair cells. A surface preparation of the cochlear epithelium isolated from a P1-P2 *Fgf8* ^P2A-FlpO*/+*^ *;Rosa26* ^*RC::RFLG/+*^ animal was stained with Alexa488-phalloidin (*green*). Native tdTomato fluorescence (*red*) is evident in most inner hair cells (i), but not in outer hair cells (o)

Founders from each of the 13 *Easi*-CRISPR targeting experiments were bred to wild type mice to transmit the mutant alleles. To date, five of the conditional and four of the knock-in alleles have produced offspring that carry the targeted alleles (Fig. 4; Additional file 1: Figure S2j–l). Of note, the biallelic founders that were bred transmitted the targeted mutation, as expected, to all offspring in their litters (Fig. 4c, *Fgf8* ^P2A-FlpO^; Additional file 1: Figure S2l, *Ppp2r2a* ^*flox*^). To determine whether the conditional alleles could be deleted using a tissue-specific Cre, we bred *Ambra1* founders to a CD4 Cre driver line [29]. Genomic DNA isolated from the peripheral blood of floxed heterozygote and *Cre*-positive offspring showed the expected recombination pattern (Fig. 5a, b). Similarly, to determine whether the knock-in alleles express as desired, one *Fgf8* ^P2A-FlpO^ founder and one *Slc26a5* ^P2A-FlpO^ F1 were bred to a FlpO reporter line [30] and the offspring were analyzed for expression of tdTomato. As expected, the offspring of these two animals showed appropriate expression of the inserted sequence (*Fgf8* ^P2A-FlpO^ #4 drove expression in cochlear inner hair cells; Fig. 5c). These results indicate that *Easi-*CRISPR can efficiently insert sequences that encode and express reporters, recombinases, and regulatory proteins, and that the technique is applicable to multiple genomic loci.

## Discussion

### Development of a high efficiency method for creating conditional and insertion alleles

Even though the CRISPR system has led to many paradigm shifts in animal transgenesis [31–33] and is routinely used to generate mice with small insertions and deletions, until now there have been no simple strategies for efficient and targeted insertion of long sequences via direct zygote microinjection of CRISPR components. There has been an intensive effort made by the community during the past 3 years to use CRISPR-based strategies for developing floxed models through zygote injections [11, 12, 17], and also through ES cell targeting strategies [20, 24, 25]. The primary objective of this work was to develop a CRISPR targeting strategy suitable for both high- and low-throughput generation of floxed animal models. The criteria we set for the new strategy were that it should be: (1) easy to design and build floxed-donor DNA cassettes, (2) compatible with zygote injections, (3) very efficient, and (4) generalizable to many loci.

To this end, we extended our previous finding that ~400-base ssDNAs serve as efficient donors for HDR at single Cas9 cleavage sites [26], by first showing that long ssDNA donors with short homology arms can be used to replace a gene segment between two Cas9 cleavage sites, a critical technical necessity for generating floxed alleles. Given the multitude of potential undesired products that are possible from the NHEJ repair pathway acting at two Cas9 cleavage sites, the high frequency of recovering correctly floxed alleles at seven different loci by using *Easi*-CRISPR (8.5–100%) was surprising, as previously described strategies reached a maximum of 16% efficiency [10, 11]. Of note, a floxed allele for *Col12a1*, one of the loci targeted here by *Easi*-CRISPR, was recently developed by using a strategy called PITCh (Precise Integration into Target Chromosome) [34]. The targeting of *Col12a1* by PITCh used the same set of ctRNP complexes that were used in *Easi-*CRISPR (described above) but the difference between the two methods was in the donor DNA format, unlike the ssDNA donor used in *Easi-*CRISPR, the PITCh system used a dsDNA donor. The PITCh approach required 265 zygotes whereas *Easi-*CRISPR used only 105 zygotes, and the PITCh approach produced 33% correctly targeted pups, whereas *Easi-*CRISPR (using ssDNA donors) produced 100% correctly targeted pups. Of note, the PITCh experiment included the Exo1 nuclease, an enhancer of targeted insertion [34]. In its absence, the efficiency would likely have been lower than 33%.

While developing our method, an IMPC study investigated whether floxed gene-targeting vectors that had already been created for ES cell targeting could be modified and used as dsDNA donors for zygote injections [35]. Although this study showed the feasibility of the approach for one gene (six targeted out of 17 live born, 35% efficiency; or one targeted out of two live born, 50% efficiency), when the same strategy was applied to two other genes, only one of the two genes yielded a floxed allele (one targeted out of nine pups for one gene, and zero out of nine pups for the second gene: 11 and 0% efficiency, respectively). In comparison, *Easi*-CRISPR offers better options for several reasons. First, the complex gene-targeting vectors must be modified in the IMPC approach before they can be used for zygote injections, whereas *Easi*-CRISPR donor designs are simple and cassettes can be synthesized rapidly by commercial custom gene synthesis services. Second, the efficiency of the IMPC method is lower for creating conditional alleles (0 to 50%), compared to *Easi-*CRISPR, which has an efficiency of 8.5–100%.

In addition to floxed alleles, knock-ins of coding sequences for recombinases and reporter genes are also critical tools for Cre-*LoxP*-based mouse molecular genetics, and they serve many additional purposes in biomedical research. Furthermore, in some studies, gene expression or site-specific recombination is achieved by using inducible systems, such as those involving the tetracycline-induced transactivators and repressors [36]. By successfully targeting insertions to six loci we demonstrate that *Easi*-CRISPR is suitable for generating all such knock-in models. Therefore, *Easi*-CRISPR offers a comprehensive solution to the Cre-*LoxP* mouse genetics system because it also allows creation of reporter/recombinase/transcriptional effector knock-ins, not just conditional alleles.

Some potential limitations of our method are: (1) Targeting single exon genes or genes containing unusually repetitive sequences. This limitation, however, is applicable to any gene targeting approach. (2) Targeting insertions longer than 2 kb. Although many types of commonly used mouse models such as floxed, Cre-, rtTA-, and reporter- knock-in alleles can be created using ssDNA donors of 1 to 2 kb long, expanding the synthesis capability of donors to several kilobases long would enable creation of a wider range of animal models. This will require technical improvements that extend the lengths of ssDNA donors. (3) Variability of cleavage efficiencies of guides (discussed below) can influence insertion efficiencies at different loci. This issue is inherent to any CRISPR-based targeting approach. We are confident that future advances to, or further modifications of, *Easi*-CRISPR will address such potential limitations.

### *Easi-*CRISPR is highly robust and generalizable

*Easi*-CRISPR is robust because one or more correctly targeted animals can be generated by injecting as few as 50 zygotes per gene-targeting project (13 targeting projects were completed using 639 zygotes). The method is also highly generalizable because it has worked for over a dozen loci. We noted a wide range in the frequency of insertions for different loci (8.5–100%). There are several possible explanations for this finding. First, we think that a major factor contributing to the differences in insertion frequencies may arise from the differences in cleavage efficiency of guides. A recent report described a systematic analysis of guide features and identified the parameters that contribute to variability in cleavage efficiency. They found that cleavage depended on many factors, including nucleotide sequences at both PAM-distal and PAM-proximal regions of the sgRNA, the genomic context of the targeted DNA, the GC percentage, and the secondary structure of sgRNA [37, 38]. It is possible, therefore, that some guides may not work and trying alternative guides for those loci may be necessary [39]. Finding a suitable, high efficiency, guide can be a critical factor for targeting experiments in which insertion of a fusion cassette at a specific codon in the genome is required and good guides are not available for the target site. In contrast, guide location is not a major factor in designs for floxed alleles because the position of *LoxP* sites in introns is flexible. For successful floxing, however, both guides need to be equally efficient at directing cleavage; if one site is cleaved less efficiently than the other, the overall targeting efficiency may be lower. This situation may have occurred with our *Pitx1* and *Syt1* targeting in which only one of the two *LoxP* sites was inserted in some animals (40% of *Pitx1* founders; 25% of *Syt1* founders). We suggest that these partial insertions occurred because the second guide may not have cleaved the genome in those zygotes. A second explanation for the variability in insertion efficiencies may be differences in the genomic loci themselves. It was proposed previously that HDR varies widely from locus to locus [40–42]. A third explanation could be the inherent variability in the experimental steps of animal transgenesis, such as embryo isolation and ex vivo handling for microinjection, variability in the embryo transfer procedure after microinjection, etc. Despite these potential limitations, the method presented here efficiently generates at least one correctly targeted animal for each locus and, frequently, most of the animals born contain the targeted allele.

### *Easi-*CRISPR will be simple to adapt for both low-throughput and high-throughput labs

Microinjection of mouse zygotes is a standard technique and with the exception of one locus (*Col12a1*; 3.8%), the birth rates of *Easi-*CRISPR manipulated zygotes were 9–31%, which is similar to that observed with conventional pronuclear injections [43]. Although these birth rates may seem low for some loci, transgenic experiments involve many complicated steps, variation in which affects birth rates. These include the quality of microinjection reagents, the many steps of assisted reproduction, animal husbandry conditions, and, finally, the mothering ability of the recipient females. Very importantly, the proficiency of technicians involved can be another major factor. For example, the extent of trauma caused to the embryos by the volume of liquid injected and the success of embryo transfers to the oviducts can vary from technician to technician. Consequently, most transgenic core labs that rely on currently available HDR strategies typically inject about 200–300 or more zygotes to generate knock-in models. Even so, in many cases, these projects are unsuccessful. Despite the factors discussed above, *Easi*-CRISPR clearly stands out as we have thus far successfully created founders for 13 loci by injecting only 639 zygotes. The majority of these *Easi-*CRISPR targeting projects were completed by injecting only about 40–60 zygotes. Of note, the founders for these alleles were created at three independent facilities by different technical staff and all projects were successful. Thus, *Easi*-CRISPR should be easily adaptable to most low- and high-throughput applications.

### Mechanistic thoughts about the high efficiency of *Easi-*CRISPR

Our results suggest that long ssDNAs are key to achieving high HDR efficiency in CRISPR genome editing. The other most significant factor contributing to the high efficiency of *Easi*-CRISPR could be the ctRNP delivery of the targeting components. Our experiment directly comparing *Cas9*-mRNA/sgRNA injection versus ctRNP (crRNA + tracrRNA + Cas9 protein) for floxing *Pitx1* showed that ctRNP delivery was about three-fold more efficient than when all components were delivered as RNA. A similar observation was reported by Aida et al. [28], who compared the sgRNA/*Cas9* mRNA, sgRNP, and ctRNP platforms programmed with dsDNA donors and concluded that ctRNP (referred to as cloning-free CRISPR/Cas) was the most efficient. A recent study of sgRNP electroporation also indicates its superior performance over sgRNA/mRNA delivery [44]. Taken together, we conclude that crRNA + tracrRNA (instead of sgRNA), Cas9 protein (instead of *Cas9* mRNA), and long ssDNA donors (instead of dsDNAs) are central to obtaining consistently higher success in CRISPR animal genome engineering. The high efficiency of *Easi-*CRISPR could also be combined with electroporation-based delivery methods such as GONAD [45, 46], TAKE [47], and SLENDR [48] to generate floxed or knock-in mice.

The unexpected observation that long ssDNA donors drive high insertion efficiencies leads to the question of why there is such a large difference between the targeting efficiencies of ssDNA versus dsDNA donors. Among different HDR types, classic homologous recombination (HR) uses dsDNA as a donor, while some recently identified processes, including single strand annealing (SSA) [49] or micro-homology mediated end joining (MMEJ; also known as Alt-EJ) [50], rely on the availability of annealable-partner sequences within the non-recessed ends themselves. Considering the properties of our donors, we speculate that the proteins responsible for either SSA or MMEJ may be involved in ssDNA donor-mediated repair. Of these two, MMEJ factors are less likely because this mechanism relies on very short homologies [50, 51]; whereas SSA operates with arms typically longer than 30 bases (the arms in our ssDNA donors are 55–105 bases) [49]. It was recently proposed that the MMEJ mechanism applies when the donors for CRISPR editing contain 5–25 base homology arms, although the protein factors involved in this repair process need to be validated. Many protein factors involved in various types of HDR pathways have been characterized [49, 51, 52] and a systematic analysis of *Easi-*CRISPR frequencies, in the absence of some of those factors in mouse embryos, will help delineate the molecular mechanisms involved.

### Other potential applications of *Easi-*CRISPR

Our results suggest that it is possible to create gene-replacement models, as demonstrated by our finding that two cleavages can be used to take out a target exon and replace it with a floxed exon cassette. Thus, *Easi*-CRISPR will also be suitable for generating other types of DNA replacements, such as (1) a set of point mutations spread across a region (e.g., up to 1–2 kb long that can be efficiently inserted), (2) testing regulatory sequences, and (3) replacing short stretches of gene segments or coding sequences from other species (e.g., creating humanized mice). In addition, *Easi-*CRISPR could be used to modify existing knock-in alleles, for example, by inserting an frt-stop-frt cassette into an existing lox-stop-lox-controlled gene to enable dual recombinase control of that gene without having to start from a wild-type allele.

Because of the availability of numerous genome engineering tools developed during the past four decades, the mouse has become the main species used to model human genetic pathophysiology. However, there are many cases in which mouse models do not recapitulate human disease and other species are preferred. *Easi-*CRISPR, with its simple design requirements and high efficiency, may provide the solution to engineering the genomes of medically relevant laboratory animals as well as livestock species for which zygote injections can be performed successfully. For example, there is a particular need for rat models [53, 54]. The community has begun exploring CRISPR strategies for generating Cre-*LoxP* rat models [15, 55] and many commercial service providers have initiated rat genome modification services [56]. We anticipate that because of its numerous benefits, including simplicity of design, high efficiency, effectiveness for many genes, and suitability for both low- and high-throughput laboratories, *Easi-*CRISPR will serve as an effective means of rapidly building mouse Cre-*LoxP* resources, and for building similar resources for rat and other models in the future.

## Conclusions

Conditional knockout and transgenic/knock-in models expressing reporters or recombinases together constitute over 90% of genetically engineered mouse models created routinely. Although it was previously claimed that the CRISPR/Cas9 system could be readily used for developing such models, it has proven to be highly challenging because the insertion of foreign DNA cassettes at Cas9 cleavage sites is inefficient. The *Easi-*CRISPR strategy we describe here uses simplified CRISPR tools; long ssDNA donors and ctRNPs, and allows the insertion of DNA cassettes into genomes with a very high efficiency. The method has been used at over a dozen loci revealing robustness, high efficiency and, moreover, versatility as it can create conditional as well as recombinase, reporter, and transcriptional effector knock-in alleles. The method is also easily adaptable to both low- and high-throughput genome engineering applications. *Easi*-CRISPR therefore solves a major challenge in the CRISPR animal genome engineering field and offers a comprehensive system for building large-scale Cre-*LoxP* animal resources.

## Methods

### CRISPR reagents

CRISPR guide RNAs were designed using CRISPR.mit.edu, or CHOPCHOP, and were used as annealed two-part synthetic crRNA and tracrRNA molecules for all genes (Alt-R^TM^ CRISPR guide RNAs, Integrated DNA Technologies, Inc. (IDT), Coralville, IA, USA and Genome Craft Type CT, FASMAC, Kanagawa, Japan), and as sgRNAs for *Pitx1*. *Cas9* mRNA (used for the *Pitx1* floxing experiment; Additional file 1: Figure S1) was prepared using the pBGK plasmid as described previously [27]. The sgRNAs (used for the ﻿*Pitx1*﻿ floxing experiment; Additional file 1: Figure S1) were synthesized as described previously [26]. The plasmid was linearized with XbaI and used as the template for in vitro transcription using the mMESSAGE mMACHINE T7 ULTRA kit (Ambion, AM 1345). Recombinant Cas9 protein employed for RNP injections was the Alt-R™ S.p. Cas9 Nuclease 3NLS (IDT), or from New England Biolabs, or FASMAC. dsDNA templates for floxing experiments (containing the homology arms and the floxed exon sequences) for producing ssDNA donors were custom synthetic genes made by Life Technologies or IDT (for floxing experiments) and knock-in cassettes were amplified using long primers to add homology arms. The ssDNA HDR donors were prepared from these cloned dsDNA templates either using the *Iv*TRT method as described previously [26] or obtained from IDT (Megamer™ single-stranded Gene Fragments). Both *Iv*TRT and IDT Megamer™ ssDNA preps showed comparable HDR efficiencies. Although the two different versions of ssDNAs have not been tested on the same genetic locus, we do not anticipate any performance differences between the two sources (Tables 1 and 2).

### Preparation of CRISPR injection mixes

The ctRNP mixes were prepared as follows. Lyophilized crRNA and tracrRNA (commercially procured) were re-suspended in microinjection buffer (TrisHCl 10 mM, pH 7.5, EDTA 0.1 mM). Five micrograms of crRNA (5 μl of 1 μg/μl) and 10 μg of tracrRNA (10 μl of 1 μg/μl) were combined in a PCR tube and were annealed in a thermocycler (95 °C for 5 min followed by ramp down to 25 °C at 5 °C/min). The annealed crRNA and tracrRNA (also known as guide RNA) were diluted in microinjection buffer and mixed with Cas9 protein to obtain ctRNP complexes [57]. The final concentrations of components in ctRNP preparations were 5–20 ng/μl of guide RNA (if two guides were used, each guide was at 5–20 ng/μl) and 5–50 ng/μl of Cas9 protein. The ssDNA donors were mixed with ctRNP complexes at 5–10 ng/mix and the final injection mixes were passed through Millipore Centrifugal Filter units (UFC30VV25, EMD Millipore, Billerica, MA, USA) and spun at 21,000 g for 5 min at room temperature.

### Microinjection of one-cell embryos

All animal experiments performed were approved by the respective institutional IACUC protocols. C57BL/6 mice at 3–4 weeks of age (Charles River Laboratories, Wilmington, MA, USA or CLEA, Tokyo, Japan) were superovulated by intraperitoneal injection of 5 IU pregnant mare serum gonadotropin, followed 48 h later by injection of 5 IU human chorionic gonadotropin (both hormones from National Hormone & Peptide Program, Torrance, CA, USA). Mouse zygotes were obtained by mating C57BL/6 stud males with superovulated C57BL/6 females. One-cell stage fertilized mouse embryos were injected with 5–50 ng/μl Cas9 protein (or 10 ng/μl of *Cas9* mRNA; for *Pitx1* locus), 5-20 ng/μl of annealed crRNA and tracrRNA (or 10 ng/μl of each sgRNA; for *Pitx1* locus) and 5–10 ng/μl of ssDNA. Microinjections and mouse transgenesis experiments were performed as described previously [27].

### Mouse genomic DNA extraction, genotyping, and sequencing

Mouse genomic DNA was extracted from toe or ear samples using the Qiagen Gentra Puregene Tissue Kit (Qiagen Sciences, Maryland, USA) or Allele-In-One Mouse Tail Direct Lysis Buffer (KURABO, Osaka, Japan). Primers were designed to amplify the correctly targeted junctions. Genomic DNA was subjected to flanking primer PCR and internal (donor oligo-specific) and external primer PCR. The primer sequences for all 13 genes are listed in Additional file 1: Table S1. PCR reactions were performed using the Go Taq Promega Hot Start green mix (Promega, Madison, WI, USA) or PrimeSTAR HS DNA Polymerase (TaKaRa, Shiga, Japan). The amplicons were separated on a 1–3% agarose gel. The gel-purified amplicons were subjected to sequencing using one of the PCR primers and/or internal primers. In some cases, PCR products were cloned into TA (Life Technologies, catalog number K2020-20) vectors before sequencing.

### FlpO activity assay

A homozygous FLP reporter mouse, B6.Cg-*Gt(ROSA)26Sor* ^*tm1.3(CAG-tdTomato*,*-EGFP)Pjen*^/J (JAX Stock #026932) [30], was crossed with the *Fgf8* ^-P2A-FlpO^ #4. Whole cochleae were dissected from P1–P2 pups, cut along Reissner’s membrane to expose the surface of the sensory epithelium, and fixed overnight at 4 °C in 4% paraformaldehyde in PBS. The cochleae were stained with Alexa488-phalloidin (Invitrogen) diluted 1:1500 in PBS containing 0.1% Triton X-100 for 15 min, and then mounted in Fluoromount-G (SouthernBiotech) on microscope slides. Cochleae were imaged on an Axioskop (Zeiss) with epifluorescent illumination and photographed with an Infinity 3-6UR (Lumenera) digital camera. Green and red channels were overlaid using Photoshop CS6 (Adobe).

### Quantification and statistical analysis

The robustness of the genome-editing method developed in this work was tested at 13 independent genomic loci. Each locus-specific experiment was performed by injecting zygotes to generate founders until at least one correctly targeted founder animal was obtained. Based on this criterion, all the 13 loci tried yielded targeted animals (i.e., 100% success rate). The number of zygotes injected ranged from 19 to 105 per locus with an average of 50 zygotes injected per locus to successfully complete a project (to obtain at least one correctly targeted animal). The overall efficiency of individual projects was calculated by the percentage of correctly targeted animals among the total number of live born animals, which ranged from 8.5 to 100%. The possible reasons of variability across different genomic loci are included in the discussion section.

## Additional file

Additional file 1: Twenty-two supplementary figures and one supplementary table. (DOCX 8910 kb)
