# Supplementary material for: Easi-CRISPR: a robust method for one-step generation of mice carrying conditional and insertion alleles using long ssDNA donors and CRISPR ribonucleoproteins
Source: Genome Biol. 2017 May 17;18:92. doi: 10.1186/s13059-017-1220-4 (PMC5434640; doi:10.1186/s13059-017-1220-4)
Supplement: Additional file 1: — Twenty-two supplementary figures and one supplementary table. (DOCX 8910 kb) [file 13059_2017_1220_MOESM1_ESM.docx]

***Easi-*CRISPR: a robust method for one-step generation of mice carrying conditional and insertion alleles using long ssDNA donors and CRISPR ribonucleoproteins**

**Additional file 1: Supplementary Figures and Tables**

**Supplementary Figures:**

**Figure. S1;** Generation of floxed *Pitx1* allele using *Cas9* mRNA/sgRNA injection

**Figure. S2;** Generation of floxed alleles for *Syt1, Syt9* and *Ppp2r2a* and their germ line breeding

**Figure. S3;** Generation of floxed *Pitx1* allele using sgRNP + two ssODN Strategy

**Figures. S4 to S10;** Schematic and the sequence of various floxed alleles; *Pitx1, Ambra1, Col12a1, Syt1, Syt9, Ppp2r2a and Ubr5* genes

**Figures. S11 to S15;** Generation of various knock-in fusion alleles of recombinases, reporters and transcriptional activators at *Slc26a5, Mafb, Otoa, Mmp9 and Mmp13* genes using *Easi*-CRISPR.

**Figures. S16 to S21**; Schematic and the sequence of various knock-in fusion alleles at *Slc26a5, Mafb, Otoa, Mmp9 and Mmp13* genes.

**Figure. S22;** Reproducibility of *Easi*-CRISPR.

**Supplementary Table:**

**Table. S1;** Primer sequences used in the study

**Figure S1**. **Generation of floxed *Pitx1* allele using Cas9 mRNA/sgRNA injection:** (**A**) Schematic showing *Pitx1* exon 2 and the location of two guides and long ssDNA donor. (**B**) Schematic of floxed allele and three independent sets of primer combinations used for genotyping of targeted insertions. (**C, D, E**) Genotyping gel images. Based on the 5‘ *LoxP* and 3‘ *LoxP* PCRs, animal #2, #3, #4, #6, contained at least one (or both) *LoxP* sites. These four samples also show thicker bands in the full length PCRs indicating two bands that could not be resolved well in the agarose gel. Upon extensive sequencing analysis of these PCR products it was concluded that only the animal #4 contained one floxed allele (*in cis*)*,* and the second allele had a deletion in its 3‘ homology arm and the other three animals contained insertions of *LoxP* sites *in trans* or only a single targeted *LoxP* site and so they were considered partial insertions. Interpretations of genotyping is summarized in (**F**); [M: monoallelic, P; partial insertion, N: no insertion]. **(G)** Microinjection details.

**Figure S2. Generation of floxed alleles for *Syt1, Syt9* and *Ppp2r2a* and their germ line breeding (A, B, C)** schematics showing the wild type alleles, floxing ssDNA donors for targeting exons 6 and 3 of *Syt1, Syt9* and *Ppp2r2a* respectively, and the corresponding floxed alleles. The lengths of ssDNA, homology arms, and the distance between the two *LoxP* sites are shown. The primer pairs and genotyping PCRs are indicated as in Figure 1F **(D, E, F)**. The floxed allele schematics show minor differences in primer locations for each gene with respect to target exon size and location. **(G, H, I)** genotyping of G0 offspring. The expected sizes of PCR amplicons (wild type or floxed) are indicated to the left of the gel images. **(G)** *Syt1* genotyping interpretation; the animals #6 had both the 5’ and 3’ *LoxP* sites, while animals #4 and #7 contained only one *LoxP* site. **(H)** *Syt9* genotyping interpretation; only the animal #12 had both the 5’ and 3’ *LoxP* sites and all others did not contain the insert. **(I)** *Ppp2r2a* genotyping interpretation; animals #1 and 2 contained one targeted allele and the second allele contained large deletion (shown by arrows) and animal #3 was a biallelic floxed animal. **(J, K, L)** genotyping of G1 offspring. The founders #6, #12, #3 for *Syt1 Syt9*, and *Ppp2r2a* were bred to wild type mice and the offspring were genotyped. (**J, K**) The arrows indicate the germline transmitted pups among *Syt1* and *Syt9* litters. **(L)** As expected, all the pups of litter of *Ppp2r2a* founder #3 were heterozygous (as analyzed by EcoRI RFLP of full length PCR)

**Figure S3**. **Generation of floxed *Pitx1* allele using sgRNP + two ssODN Strategy:** (**A**) Schematic showing *Pitx1* exon 2 and the location of two guides and two ssODNs. (**B**) Schematic of floxed allele and three independent sets of primer combinations used for genotyping of targeted insertions. Note that primers 5’ LoxF and 3’ LoxR bind partly to *Pitx1* genomic locus and partly to the *LoxP* sites. (**C**) Genotyping gel images. All animals containing expected sized amplicons are indicated with asterisks (animals #2, #5, #7 and #14 for 5‘ *LoxP* and #2, #6, #8 and #12 for 3‘ *LoxP*) and those containing shorter bands (suggestive of deletions) are indicated with arrows (animals #2, #5, #8 and #16 for 5‘ *LoxP* and #2, #5, #14 and #16 for 3‘ *LoxP*). The results from all three PCRs, together, suggest that only animal #2 contained both *LoxP* sites on one allele and a deletion allele (possibly mosaic). (**D**) Sequencing chromatogram of the 5‘ *LoxP* site of animal #2 (the only sample out of 18 live born that contained both *LoxP* sites) shows a two nucleotide deletion in its 5‘ *LoxP*.

**Figure S4.**

1. **Schematic and the sequence of *Pitx1* floxed ssDNA. Various sequence elements are color-coded. The primers used for amplifying *LoxP* insertion sites and their amplicon sizes are shown above the schematic. Genotyping primer sequences are underlined.**

**Wild Type: 730bp; *Pitx1* floxed: 764bp**

**Wild Type: 568bp; *Pitx1* floxed: 602bp**

***Pitx1* 3’- F**

***Pitx1* 5’- R**

***Pitx1* 5’- F**

***Pitx1* 3’- R**

**Flanking Sequence**

**Right Arm**

**Flanking Sequence**

**Left Arm**

**Intron**

**2**

**Intron**

**GAAATGGCCATCTGACTGGAGTTTATACACATTTGGCTGTGTGTTTAGTTTAGGGGTGTGTTCACAGGAACGGTTTTGACTTCTTGATCTACATTTGCTCCGGTTCTGCCTCCCTGGCTCTGAGGGTTTGTGCACCTTTCTGTTTCTAGGGAGAGAGCTCTGAGCTTTTCTAGGGCTCCGTTTTGGTTTGTAGTTCCTTTGCGTTATGTTTGAATGCTATTGACTACTGTGTGCATCTTGAGTAGCTTTAAAGAGACAGAGAGGCCCGGGTCACTTAGACAGAATATGTCTGTCTAGGCTGCAAATGGAGGGTTTTCTAGACCTCAGAATCCGAGGCCGGTAAGGCTGGGATCCAAGCTAAGTTGCGGCTCTGACACAGCCCTGGCAAGAACAACATTCCAGGCATAACTTCGTATAGCATACATTATACGAAGTTATGACTACATGGCTCTTACCCCACAGATATGCTAGCTTCAGCTGGAAAGTTTTGATGCCCGTTCAGGAAAGCTGAGGTGTCCGCTCAGATTTAGACCTTCAAGAGTCTCAGCAGGGCCGCAGCCAGCGAGCTGCAGAAGCAGGCCCTGCCTAGCTGGGAACCCAGGCCCAGATGTGGCTCCCGGGCTCTTTGCTGTGGCACGCCAGATCTAGGGGTGCTTACTGAGAACCGGCACCCGGCCCTTCCTTTCCAGCGGGGAAACAGTTCCCAGGGCCCCTGGATGGAGGGAGGGAAGCCACCGCTGACGGCAGGCGCTTCGGTGGAGCCGGGACCGGGCGCGATAGTGTTAGCCCGAGGTGACTCGCGCCCATTCCTACCTGCTGCAGACAAGGAGCGCGGTGGGGAAGCCAAGGGGCCAGAGGATGGTGGCGCGGGCAGTGCTGGCTGCGGCGGCGGTGCAGAGGACCCAGCTAAGAAGAAGAAACAGCGGCGGCAACGCACTCACTTCACAAGCCAGCAGTTGCAAGAGCTGGAGGCCACGTTCCAAAGGAACCGCTACCCCGACATGAGCATGAGAGAGGAGATCGCGGTGTGGACCAACCTCACTGAACCGCGAGTGCGGGTGAGCGTGGGGCGCGTGCAGAGGGCTACCGGCTGGCACTCAGTAGCTTAGACTTACAACTCTGACCTCGCGGACCTTGCTTCGTAGGAGACCCCACTTTAGGCCCCAGCCAGCCACCCACGTCAGCCAGGTCCCTGGGCTACTGCTTTCTTAGCCCTTTTACCTGTCCCACAGATAACTTCGTATAGCATACATTATACGAAGTTATGAGCTACTGCAAGCCCCTGTCGGAAGACCCTGTCCCTGCCCTGAACTCTGTCAGAATTACTCAGAGCCGGAGATTGGGCAGAGGATTCTCTGCCGCTAAGGACCTCGGGAGCTTTAAGCCTTCATCCCCTGGAAAACGAAAGGGAAAGACAGGTCGTGCCTGGCTGCCTCAGAGAGGCCTGAAAGGTGCTTGAGTTGGGGTATCTCAAACTTCTTGGAACTGTGATCTCATTCTGAATTTCCCTTCTCCAAACTGTTTTTGGAATATTCTCCATCTTTTCTGGCCAGTCAGCCAGGTACTAGCTGGACCTTTGCCTAATGCCCTTTCCCACTGTAGGTCTGGGGTCATGTGGA**

1. **Sequencing of the *LoxP* insertion sites in a founder G0 pup showing correct insertion of the *LoxP* sites The PCR amplicons were sequenced using one of the respective PCR primers.**

**Figure S5.**

1. **Schematic and the sequence of *Ambra1* floxed ssDNA. Various sequence elements are color-coded. The primers used for amplifying *LoxP* insertion sites and their amplicon sizes are shown above the schematic. Genotyping primer sequences are underlined.**

**Wild Type: 502bp; *Ambra1 floxed*: 536bp**

**Wild Type: 399bp; *Ambra1* floxed: 433bp**

***Ambra1* 3’- F**

***Ambra1* 5’- R**

***Ambra1* 5’- F**

***Ambra1* 3’- R**

**Intron**

**4**

**Right Arm**

**Flanking Sequence**

**Flanking Sequence**

**Left Arm**

**Intron**

**TTGGCTTAGCTTGTTCATTCAGATTAATCCCTAGAGACCCTTTGAAGCACTGGATGAATGTGTGAAGGTGTATATGGTGTACACACACACACTCTATTATTTATAATTATATAATTATATAATTATATAATTATATATATATATATATATATATATATAATTATACTATATATTAGAAATGGAGAGAAAGTAAACAAACAATGCCAACCAGAAGAATAAAAAGAATTGTAAAGAGAATGGTGAAAGTCAAGGCTCAGAAGATAACATTGCTTTGCTTGACCAGTAATAACTTCGTATAGCATACATTATACGAAGTTATGACACCAGCAATGAAGGCAATATGTGTTATTTAGCGACAGTGTGGGAAAACCAGAAGCCTTTAAAAATGGGAATATTTCTGTTTTGTGTAGAGAAACTACCTAGTAGGCATACCTTAATTTTTTTTCATTTGTAGTGAGTAGTGTAGGGAATAATGTTGGGATTTTTGTTTTCTAAAGCTGGTAATATTTATTCTCCCCCTACATGCTTCTTAATTATTCCTACATTTTGATTCTCTAGGACTCTCTTGGCATCCACACATGTGAACCATAACATCTATATTACAGAGGTTAAGACTGGCAAGTGTGTTCACTCTCTGATCGGACACCGCCGTACTCCATGGTGTGTCACTTTTCACCCCACCATCTCAGGCCTTATTGCTTCTGGTTGCCTAGATGGGGAAGTTAGGATTTGGGATTTACATGTAAGTATTTTTTGGGCTGCTTATTTCCCTTTGGTATTCCAGACACAGAGACTTGTTTGCCATTGGATTCTTACTTCTGGCTGAGAAACTAACCACTTATAACTTCGTATAGCATACATTATACGAAGTTATTGTTAGGACTGGTAGACATTGCTTACTTAAAGGATGGATATTCTAGTTTGTACTTATCCATTATGAAGTAAACAAACTTTTCTTCTGCCTGCTCATTTTAAGATTGTATAGCAGGAGGCTCTAACATGAGTTCAGTATCGACTTCCTTTGTCTTGGAGTACTCTGCTCATGATCATCACACAAGACTTGTCCTGATGATAGACTAGTGGGTTGCTTTCACTATGTTTGCACCAAGCTGGAATGTTCACAGGTGTCAGAAACAGGAACTAAGTAGAGGCTTTGTAATTGACTTGAAGACAGTAATTGTTCTATAAAT**

1. **Sequencing of the *LoxP* insertion sites in a founder G0 pup showing correct insertion of the *LoxP* sites The PCR amplicons were sequenced using one of the respective PCR primers.**

**Figure S6.**

1. **Schematic and the sequence of *Col12a1* floxed ssDNA. Various sequence elements are color-coded. The primers used for amplifying *LoxP* insertion sites and their amplicon sizes are shown above the schematic. Genotyping primer sequences are underlined.**

**Wild Type: 403bp; *Col12a1* floxed: 443bp**

**Wild Type: 449bp; *Col12a1* floxed: 483bp**

***Col12a1* 5’- R**

***Col12a1* 3’- F**

***Col12a1* 3’- R**

***Col12a1* 5’- F**

**2**

**Flanking Sequence**

**Right Arm**

**Intron**

**Intron**

**Left Arm**

**Flanking Sequence**

**TCACCTTGGCAGTATGAAGTCATGTGCGGTCTGGTCAGGCCTTTACAGGGCACCAGGCAGCAGCAGCTCCCTGGGGGAAGAATATTCTCTTTAAACTAGAAT** **TTCCTACTAGTGCCCTGATTAAACCTATTGGAAGAGCTTGACTTCCATGGTTCCAaagcttATAACTTCGTATAGCATACATTATACGAAGTTATCAATGGGTCCATTATGTGCTGGGCTGGGCCCTGTTGTTTTATAGGCAGATTTGGCTCGGTTCTGGTCCATGTTTAGGCTCTAGTCTAACCTTGTTCGTGGCCTGCTCAGGCTGGGTTCTGATGGATCACTGCTTCCTCCTGCAGATCCCTTCATGAGGCTGTGAGCTTGAGGCCCAAACATGCAGACCAGGCTTCCCCGAGCGCTGGCCGCCCTGGGCGTGGCCCTACTCCTGTCTTCCATTGAGGCAGAAGGTAAAATGCTTTTACTTCTCCTATGACAAGTGAAACTGCAGAGTTCAAGAAAGGAAAAGACTTTCATCTCCTGCTTCTGTTGCTTGGTCTGTTTTCTCTGCACTTCTCAGGTGCTTTCTCTTCTTCAGAGAAACACCTGCCTTCTGAGGTGTGTCCGTTTCTGAACTTTAAAGGTCCAGCTCGTTTATTCTGCTTCCACTAATAACTTCGTATAGCATACATTATACGAAGTTATTTCTGTACAGTGCTGTGTGGTCTCCTTGCGCTTGTCTCTGCAGTGTGCTGTCCACACTGCCACCTGCACTGCCGCCTTCCTTGACGATCAGTCCCCTGGATAGGGCAACAACTGTATCCAGCTCTGACAAGAGGATTCCCGACATCTGGACACCAGGAACCTCAGAATATGGACACCTTTACAGGATCAGCAGCGGATCACCTGGGTCAGGACTGTGACCTTGGTGACAACATGAAATAAAAGACTCCAACACTGCCAGCCCCGAGCGTCTGGTTTTCCTTTCTTTTCTTTTTGAGCAGATTAGGTTGGGTAGGCAG**

**(b) Sequencing of the *LoxP* insertion sites a founder G0 pup showing correct insertion of the *LoxP* sites and the HindIII site. The PCR amplicons were sequenced using one of the respective PCR primers.**

**Figure S7.**

1. **Schematic and the sequence of *Syt1* floxed ssDNA. Various sequence elements are color-coded. The primers used for amplifying *LoxP* insertion sites and their amplicon sizes are shown above the schematic. Genotyping primer sequences are underlined.**

**Wild Type: 418bp; *Syt1* floxed: 458bp**

**Wild Type: 484bp; *Syt1* floxed: 524bp**

**Syt1 5’- R**

***Syt1* 3’- F**

***Syt1* 5’- F**

***Syt1* 3’- R**

**Right Arm**

**Intron**

**6**

**Intron**

**Left Arm**

**Flanking Sequence**

**Flanking Sequence**

**GCCAGCTTTATCTCTATTGAATAGACGAGGAGGCAGAGGCAGAGAGGAGATTCGTACAAGATGGCAGAGCTGGGATACAGTGAAACTAGAAAACTGCCTGGCAAGCGACGTGTAAATGAAGAGTTAATAGGAAATTAGCTTGGATCCTATGGGAAAGGTGGCATGTAGTATACAATAACTTCGTATAGCATACATTATACGAAGTTATAAGCTTTAGAGGTAGAAGAAGAGTCCAGGCCAGGTCACAGCACTGATAAAACCCTACAGATCAGTACCACTGGAGCAAAACAAGCGCAGGGGAAACCCAGCCCCAAGCATGGATGTCTTGGCTGCATTTGGTTAACTGGTCCACTGCTGATGTTCCGTTTCTCTCTCTCTCTCTTCCAGTGCCACCGTGGGCCTTAATTGCCATAGCCATAGTTGCGGTCCTTCTAGTCGTGACCTGCTGCTTCTGTGTCTGTAAGAAATGTTTGTTCAAAAAGAAAAACAAGAAGAAGGGAAAGGAAAAGGGAGGGAAGAACGCCATTAACATGAAAGACGTGAAAGACTTAGGGAAGACCATGAAGGATCAGGTAACGCACCGGCCCCCTCATCCCCATTCACAGAGGTCCCTGAGAATTCACCAGTGGGGCTGTGCTCTTTTCTGTAGATGCTTATAGACATGGACAACTTGGGGCTACTGTTGTTTTTGTAGAGATGCTTGGCGGTCCCTTCAGGTATGACCCGGTCTCCCAAAGTGTAGTGGAGACAGGTACGGTGGCTTCCACACAAGCTTATAACTTCGTATAGCATACATTATACGAAGTTATAACGAGGTGTGGCTTCACGGACCTGGCATAATAAGATAATTCCCGAACATTTTTATTCCGTTTTTTGTCATCCAGTCTTTCTGTCTTGGACAGGGGTTCTTTCTGCCTCCTGCACTGTTCTTAGTCTCTGTTAAAGCTCTTCATCCACTTGGCAATGCCAGCACTGCTCTGCACCCGGCCAGCTCTGCCAT**

**(b) Sequencing of the *LoxP* insertion sites a founder G0 pup showing correct insertion of the *LoxP* sites and the HindIII site. The PCR amplicons were sequenced using one of the respective PCR primers.**

**Figure S8.**

1. **Schematic and the sequence of *Syt9* floxed ssDNA. Various sequence elements are color-coded. The primers used for amplifying *LoxP* insertion sites and their amplicon sizes are shown above the schematic. Genotyping primer sequences are underlined.**

**Wild Type: 617bp; *Syt9* floxed: 657bp**

**Wild Type: 654bp; *Syt9* floxed: 694bp**

**Syt9 5’- R**

***Syt9* 3’- F**

***Syt9* 5’- F**

***Syt9* 3’- R**

**Intron**

**3**

**Right Arm**

**Intron**

**Left Arm**

**Flanking Sequence**

**Flanking Sequence**

**ATATGTAGCAGAGGTTGGCCTTGTCAGGCATCAATGGGAGGAGAGGTCATTGGTTCTACGAAGGCTTGATAGGTGCCCCTGTGTAGGGGAACCCAGGGCGG GGAGGTGGGAGTGGATGGGTGGGTGGAGGAACACCCTCATAGAAGCAGGGGGAGGGAGGATGGGATGGGGTTTCTGGGAGGGGGCAAATAACTTCGTATAGCATACATTATACGAAGTTATGAATTCAACTGGGGAAGGAGATAACATTGGAAATATGAATAAAGGAAATATTAAAAAAGAAATAGAACAGCGCACTGACATTGCAGACACAAGGTTCTGAAAGACGGGCAGCTCATGAGGTTTTAACTCTGCTGTGTCTTGCTCTTCGTTTAAGGCATAACTCAATCCGAAGACAGCTCAACCTGTCGAACCCGGACTTTAATATCCAGCAGCTTCAGAGGCAGGAGCAGCTGACTGGGATTGGTAGAATTAAACCAGAGTTATACAAACAGAGGTCACTGGACAACGACGATGGGCGGAGGAGTAACAGCAAAGCCTGCGGGAAACTGAACTTCATTTTAAAATATGACTGCGACTTGGAACAGCTCATTGTGAAGATCCACAAAGCCGTCAATCTGCCTGCCAAGGACTTCTCTGGGACTTCAGATCCTTATGTCAAGATCTACTTGCTTCCTGACCGGAAAACAAAACACCAGACTAAAGTTCACAGGAAGACCCTGAACCCTGTGTTTGATGAGGTGTTTTTATTTCCTGTTCACTACAATGACCTTGAAGCTCGGAAGCTTCACTTCTCTGTGTATGACTTTGACAGGTTCTCTCGCCATGACCTGATTGGTCAGGTGGTGGTGGACCACTTCTTCGACTTGGCCGACTTCCCCAGGGAGTGCATCCTTTGGAAGGATATCGAGTATGTCACCAACGTGAGTCTAGCCTTCTTCCATTGGGCTGGGGGAGTTGCTTCTTCTTTTCCACAGAAACAGACTGCTTACACGTGTTATTTGGGAACAAACGCTAAAATTCACATTGATATCCTATCTTTGGAGGAATTCATAACTTCGTATAGCATACATTATACGAAGTTATGGTGGGTTTTTAATAGCATTAACTTTGATACATACAAGAATAATTTGCTAACACTATTAAGAAATACCTTTTTTTTTTTTTTTTTTTTTGCTTTTCTTCTATCATAGCTGCTCACAACATTTTCTTGAGAGCTGTTTCAGGTTTGTATTAACTGGGTCATGGCATCCTGTTCA**

**(b) Sequencing of the *LoxP* insertion sites a founder G0 pup showing correct insertion of the *LoxP* sites and the EcoRI site. The PCR amplicons were sequenced using one of the respective PCR primers.**

**Figure S9.**

1. **Schematic and the sequence of *Ppp2r2a* floxed ssDNA. Various sequence elements are color-coded. The primers used for amplifying *LoxP* insertion sites and their amplicon sizes are shown above the schematic. Genotyping primer sequences are underlined.**

**Wild Type: 403bp; *Ppp2r5a* floxed: 443bp**

**Wild Type: 496bp; *Ppp2r5a* floxed: 536bp**

***Ppp2r2a* 3’- R**

***Ppp2r2a* 3’- F**

***Ppp2r2a* 5’- R**

***Ppp2r2a* 5’- F**

**3**

**Flanking Sequence**

**Right Arm**

**Left Arm**

**Flanking Sequence**

**Intron**

**Intron**

**GACTTTCACGAAGGAAACTAGTTATGAGGAGTTCAAGAGTATCCCAGGCTACAAGAGACCACTTTTCCAAACAACCCACCAACTGGGCTTTCAGGATGGTTCAGTGTTTGTAGTGCAAGCCTGGAAACTTATGTGAAGATGAAGAGAACCTGTTCTACAAACTTGCCCTCTGACCTCTACATGCACCTGTTGGCTTAGTCCCCCTTTTACAAGTCACACATATACATGGTTATAATAGCAAATCCCACAAATAAGTGTCTGGTTTCTTTTTTTCATTCACAAATTAGTGAAATATTTACAAACCTGGCATTTATTATTATCTTCCTGATATAACTTCGTATAGCATACATTATACGAAGTTATGAATTCTGGTTGAATGGCTTTGAGTCAGTTTATTTGGGGGAGCCTCTCCCCCAAATGAAAGTGTTTTATGGAAATGTTTCTGATGTGCATAGATGGAGAATGCTGATATGAACATCCTTAGTGAGTTGAGGGTCAAAAACTGATGTCTTTTTGTTTTTGTTTCAATTATACAGCAGATATAATTTCTACAGTAGAGTTTAATCATTCTGGAGAATTACTAGCCACAGGAGATAAAGGTGGGAGAGTTGTCATCTTTCAACAAGAGCAGGAGGTAAGTACTGACAGTTCAGTGTTGCCTTTTCAGTTTGATCTTGAGATTGGAGGTTGTGCTGGAAAAATCCAGCTTTAACGCTGGACCCTGAGTAAATTTTTGTCTGCAGATAGCTTACGGTTTCTCTTAGCTCTGCGTGTGTGTGCTCCTGTATGTATGCCTTTGTTGCTTTTATTTGAAAACCCATTTGAATTGTAAAAGCCTTTGGTAGAGAAGAGGGTGGGAAGTAGTTGATTAATCTGAGACTGAGCCAGATTTGAAGTCTTTACCCTCAGAATTCATAACTTCGTATAGCATACATTATACGAAGTTATGTAATAAGTTGACCTCTCAAGGCTTAGCTGCTCTGTGTAATTGAGGGTAAGGTTGATTGGAAGAAAATCAGCCAAATTACTTTATAATCAGTTCATACAAAAACCATTACTAGCCTTAAATGTCTGTGTTGTGCTTGCACCTATGTGAATGTAATTAATGCATCAACACAGTGCTAGCTTTTAAAATAATTTTTTTTGGATACTGTTCCCTTTCCTCTTAAGAATGAAAAAAT**

1. **Sequencing of the *LoxP* insertion sites in a founder G0 pup showing correct insertion of the *LoxP* sites and the EcoRI sites. The PCR amplicons were sequenced using one of the respective PCR primers.**

**Figure S10**

1. **Schematic and the sequence of *Ubr5* floxed ssDNA. Various sequence elements are color-coded. The primers used for amplifying *LoxP* insertion sites and their amplicon sizes are shown above the schematic. Genotyping primer sequences are underlined.**

**Wild Type: 493bp; *Ubr5* floxed: 533bp**

**Wild Type: 405bp; *Ubr5* floxed: 445bp**

***Ubr5* 5’- R**

***Ubr5 3’*- F**

***Ubr5* 3’- R**

***Ubr5*  5’- F**

**58**

**Flanking Sequence**

**Right Arm**

**Left Arm**

**Flanking Sequence**

**Intron**

**Intron**

1. **Sequencing of the *LoxP* insertion sites in a founder G0 pup showing correct insertion of the *LoxP* sites and the EcoRI sites. The PCR amplicons were sequenced using one of the respective PCR primers.**

**CGTTGGTTGTCCTGGAACTCACTCTGTAGACCAGGCTTGCCTCGAACTCAGACATCCGCCTGCCTCTGCCTCCCAAGTGCTGGGATTAAAGGTGTGCGCTGCCCTTGCCACCACCACCCCCCAGTAGAAATGTAGACTCTTTGGTGTCCTTTTCTTCCATCTTCTAAAAGGTTTCATGTGGCTTATAGCTCAGTGGTTTGAGGAGCACTGTGGCTTAAAGTCACAGCAGAAGAAGAGGGTTTACAGATGAGGCTGACATCAACCTTGGTGGTATAACTTCGTATAGCATACATTATACGAAGTTATGAATTCGACAGGTCACTCTCTCTTACTCATCCAGAGTCAGGATGACCCAATTAATTGTACAGGTTTGTAGTTACACAAAAAGCTTTAGTTTCTTGTTAGGAATTAGATGTCTTAAATTTTAAAGTTCAATGTATCTCTGTTTATATAAGCTTACTAGTATTCAAAAGAAACAACATCTAAAACAACACATTGAATTTATATTCTCAGGAGAAAATGCTGAGAAGCTCCTCCAGTTCAAACGGTGGTTCTGGTCAATAGTAGAGAAAATGAGCATGACAGAACGGCAGGACCTGGTGAGTGAGAAGTCTCACAAAACTTGTAGAGTGAAATGCAAGGGGTCTGCTATGCTTTGTTGTTTGGTTTTGTTTGTTTTTTGGGTGTGTGTTTGTTTTCGGGGATGGGATGGTGGGTCTCTCTTGGAGAACAGTTTACGGATAAAGGTTCTGTGAAGTCTGCCACTTATAACTTCGTATAGCATACATTATACGAAGTTATGAATTCTGGTTGATCATTCATCCCACTCCTTCCCTCCTCTCTTCCATTTCTCCTGTATGTATTTTATCTGCATGTCTGTCTGTGTACCATATGTGTGTGGTGTCTTCAGAGGCCAGAAGAGGGTGTCAGATCACCTGGGCCTAGAGTTCGAGTTCTGAGTCATTGTTTGGGTCCTGGGAATGAAACCCAGAGCTTTTGGAGAAGCATCCATTCCCTGCTGACCCAGTCTCTAGCTCCATCTCAGTCTGGAATCTGTTTCCAGTAAGCCTTTTCTTCCCTGTTAACTGAGTGGATGAGTCCGGCTATGTTCCTGGTTCTTT**

**Figure S11. Fusion of a P2A-FlpO cassette to the 3’ end of *Slc26a5* gene using *Easi*-CRISPR.** (**A**) Schematic showing *Slc26a5* ssDNA donor template and targeted knock-in alleles. The lengths of ssDNA, homology arms and the P2A-FlpO cassettes are indicated. (**B**) Genotyping G0 animals for detection of cassette insertion. Schematic of primer locations for 5’ and 3’ junction PCRs is shown along with the expected amplicon sizes. Animals #1 and #3 are positive for the P2A-FlpO cassette insertion using both 5’ and 3’ PCRs. Note that the 3’ junction PCR for animal #1 is bigger than the expected size (shown by an asterisk) suggesting that it contains additional sequences (the exact sequence of the insertion was not determined). (**C**) The guide RNA sequence (italics), along with the cut site, PAM sequence (in red) and a few bases of flanking sequences are shown above. Sequencing of animal #1 indicates correct insertion of the cassette at 5’ and 3’ junctions*.*

**Figure S12. Fusion of a P2A-FlpO cassette to the 3’ end of *Mafb*** **gene using *Easi*-CRISPR.** (**A**) Schematic showing *Mafb*, ssDNA donor template and targeted knock-in alleles. The lengths of ssDNA, homology arms and the P2A-FlpO cassettes are indicated. (**B**) Genotyping G0 animals for detection of cassette insertion. Schematic of primer locations for 5’ and 3’ junction PCRs is shown along with the expected amplicon sizes. Animals #7 and #8 are positive for the FlpO cassette insertion by both 5’ and 3’ junctions PCR WT: wild type, M: 100 bp marker. (**C**) Sequencing animal #7*.* The guide RNA sequence (italics), along with the cut site, PAM sequence (in red) and a few bases of flanking sequences are shown above. Note that the guide cut site for *Mafb* is 13 bases downstream of the desired stop codon TGA (underlined). Sequence chromatograms indicate correct cassette insertion junctions.

**Figure S13. Fusion of rtTA-polyA cassette after the initiation codon of *Otoa.*** (**A**) Schematic showing the *Otoa* locus, ssDNA donor and resulting targeted insertion alleles. (**B**) Genotyping of G0 animals. Schematics of primer locations for targeted 5’ and 3’ junction PCRs are shown along with the expected amplicon sizes. Samples from *Otoa* G0 animal #2 has a correctly targeted rtTA insertion as seen by both the 5’ and 3’ junction PCRs. (**C**) Sequencing of 5’ and 3’ junctions in *Otoa* founder #2*.* The guide RNA sequence (italics), along with the cut site, PAM sequence (in red) and a few bases of flanking sequences (above) and sequence chromatograms showing correctly targeted 5’ and 3’ junctions (below).

**Figure S14: Fusion of a T2A-mCitrine cassette before the stop codon of *Mmp9.*** (**A**) Schematic showing *Mmp9* ssDNA donor template and targeted knock-in alleles. The lengths of ssDNA, homology arms and the T2A-mCitrine cassettes are indicated. (**B**) Genotyping G0 animals for detection of cassette insertion. Schematic of primer locations for targeted 5’ and 3’ junction PCRs is shown along with the expected amplicon sizes. Genotype interpretations are summarized below the gel image [M: monoallelic, P; partial insertion, N: no insertion]. Note that the junction PCR products for animals #4 and #10 contain smaller or bigger amplicons than the expected size (shown by arrow) suggesting deletion of or addition of extra-sequences, respectively (the exact sequence of the insertion was not determined). Animal #4 had a correct insertion in addition to a partial insertion. (**C**) The guide RNA sequence (italics), along with the cut site, PAM sequence (in red) and a few bases of flanking sequences are shown (above). Sequencing of animal #1 showing correct insertion of the cassette at 5’ and 3’ junctions (below).

**Figure S15: Fusion of T2A-mCherry cassette before the stop codon of *Mmp13.*** (**A**) Schematic showing *Mmp13* ssDNA donor template and targeted knock-in alleles. The lengths of ssDNA, homology arms and the T2A-mCherry cassettes are indicated. (**B**) Genotyping G0 animals for detection of cassette insertion. Schematic of primer locations for 5’ and 3’ junction PCRs is shown along with the expected amplicon sizes. Genotype interpretations are summarized below the gel image [M: monoalleic, B: biallelic, P; partial insertion, N: no insertion]. Animals #3, #6, #7, #9 are positive for the T2A-mCherry cassette insertion using both 5’ and 3’ PCRs. (**C**) The guide RNA sequence (italics), along with the cut site, PAM sequence (in red) and a few bases of flanking sequences (above). Sequencing chromatograms of animal #3 showing correct insertion of the cassette at 5’ and 3’ junctions (below)*.*

**Figure S16.** **Schematic and sequence of the *Fgf8*-P2A-FlpO-ssDNA. Various sequence elements are color-coded. The primers used for 5’ and 3’ junction PCR and the amplicon sizes are shown above the schematic. Genotyping primer sequences are underlined.**

***Fgf8* 3’- R**

**FlpO 5’- R**

**FlpO 3’- F**

**434bp**

***Fgf8* 5’- F**

**283bp**

**Flanking Sequence**

**Right Arm**

**Left Arm**

P2A

**Flanking Sequence**

FlpO

**GGACTGCGTATTCACAGAGATCGTGCTGGAGAACAACTACACGGCGCTGCAGAACGCCAAGTACGAGGGCTGGTACATGGCCTTTACCCGCAAGGGCCGGCCCCGCAAGGGCTCCAAGACGCGCCAGCATCAGCGCGAGGTGCACTTCATGAAGCGCCTGCCGCGGGGCCACCACACCACCGAGCAGAGCCTGCGCTTCGAGTTCCTCAACTACCCGCCCTTCACGCGCAGCCTGCGCGGCAGCCAGAGGACTTGGGCCCCGGAGCCCCGAGGAAGCGGAGCTACTAACTTCAGCCTGCTGAAGCAGGCTGGAGACGTGGAGGAGAACCCTGGACCTGCTAGCATGGCTCCTAAGAAGAAGAGGAAGGTGATGAGCCAGTTCGACATCCTGTGCAAGACCCCCCCCAAGGTGCTGGTGCGGCAGTTCGTGGAGAGATTCGAGAGGCCCAGCGGCGAGAAGATCGCCAGCTGTGCCGCCGAGCTGACCTACCTGTGCTGGATGATCACCCACAACGGCACCGCCATCAAGAGGGCCACCTTCATGAGCTACAACACCATCATCAGCAACAGCCTGAGCTTCGACATCGTGAACAAGAGCCTGCAGTTCAAGTACAAGACCCAGAAGGCCACCATCCTGGAGGCCAGCCTGAAGAAGCTGATCCCCGCCTGGGAGTTCACCATCATCCCTTACAACGGCCAGAAGCACCAGAGCGACATCACCGACATCGTGTCCAGCCTGCAGCTGCAGTTCGAGAGCAGCGAGGAGGCCGACAAGGGCAACAGCCACAGCAAGAAGATGCTGAAGGCCCTGCTGTCCGAGGGCGAGAGCATCTGGGAGATCACCGAGAAGATCCTGAACAGCTTCGAGTACACCAGCAGGTTCACCAAGACCAAGACCCTGTACCAGTTCCTGTTCCTGGCCACATTCATCAACTGCGGCAGGTTCAGCGACATCAAGAACGTGGACCCCAAGAGCTTCAAGCTGGTGCAGAACAAGTACCTGGGCGTGATCATTCAGTGCCTGGTGACCGAGACCAAGACAAGCGTGTCCAGGCACATCTACTTTTTCAGCGCCAGAGGCAGGATCGACCCCCTGGTGTACCTGGACGAGTTCCTGAGGAACAGCGAGCCCGTGCTGAAGAGAGTGAACAGGACCGGCAACAGCAGCAGCAACAAGCAGGAGTACCAGCTGCTGAAGGACAACCTGGTGCGCAGCTACAACAAGGCCCTGAAGAAGAACGCCCCCTACCCCATCTTCGCTATCAAGAACGGCCCTAAGAGCCACATCGGCAGGCACCTGATGACCAGCTTTCTGAGCATGAAGGGCCTGACCGAGCTGACAAACGTGGTGGGCAACTGGAGCGACAAGAGGGCCTCCGCCGTGGCCAGGACCACCTACACCCACCAGATCACCGCCATCCCCGACCACTACTTCGCCCTGGTGTCCAGGTACTACGCCTACGACCCCATCAGCAAGGAGATGATCGCCCTGAAGGACGAGACCAACCCCATCGAGGAGTGGCAGCACATCGAGCAGCTGAAGGGCAGCGCCGAGGGCAGCATCAGATACCCCGCCTGGAACGGCATCATCAGCCAGGAGGTGCTGGACTACCTGAGCAGCTACATCAACAGGCGGATCTAGGCGCTCGCCCAGCTCCTCCCCACCCAGCCGGCCGAGGAATCCAGCGGGAGCTCGGCGGCACAGCAAAGGGGAGGGGCTGGGGAGCTGCCTTCTAGTTGTGCATATTGTTTGCTGTTGGGTTTTTTTGTTTTTTGTTTTTTGTTTTTGTTTTTTGTTTTTTAAACAAAAGAGAGGCTCTATTTTTGTATTCCACTGGCTGTGGTGTCTGTCTTCTTAGCTCTCAGGAAAAGCCCACTAGTGACCTAAGACTGGGTTCCTCTGGGGTTCCCTGGGGTGGAGGGCTCTCTGGTCTGTTCTCCTGAAATCCCAGGATTGGCAGGGTACCTGGCCAGGCCAACCAGGGCCACTCTGTACCCCCGGCAGGGGGAGAGAGAGAGAGAGAGAGAGAGAGAGAGAGAGAGAGATATTAGGCCTAGGTCAGTCCTTTAGGGGAGTGGGTGTCAGTGAAGGTGGCTGTGTACCAGTTACTA**

**Figure S17. Schematic and sequence of the *Slc26a5*-FlpO-ssDNA. Various sequence elements are color-coded. The primers used for 5’ and 3’ junction PCR and the amplicon sizes are shown above the schematic. Genotyping primer sequences are underlined.**

**FlpO 5’- R**

***Slc26a5* 3’- R**

**FlpO 3’- F**

***Slc26a5* 5’- F**

**324bp**

**287bp**

**Right Arm**

FlpO

**Flanking Sequence**

**Left Arm**

P2A

**Flanking Sequence**

**ACCAGGCATAAGCCCCAGCATGTGGGAAAGGTGGTGGCAGAATTCAAGGCCAGCCTAGGTTACATAAGAACCTGTCTCTAAATTAACAAATTAAATTTTCAAGCCATAAGTGAACCGGCTTTTTTCCACTGTTTAAGACCAAAACTTGGAATGTGATTTGATTTTCTTTTCTTTGTATAGCACAAGTTGTGAATGACCTCACCCGCAACAACTTTTTTGAAAATCCTGCCTTGAAAGAGCTTCTGTTCCACAGTATCCACGATGCAGTCCTGGGCAGCCAAGTTCGGGAGGCAATGGCTGAACAAGAAGCCACAGCGTCACTTCCCCAGGAGGATATGGAGCCCAATGCCACACCCACCACCCCCGAGGCAGGAAGCGGAGCTACTAACTTCAGCCTGCTGAAGCAGGCTGGAGACGTGGAGGAGAACCCTGGACCTGCTAGCATGGCTCCTAAGAAGAAGAGGAAGGTGATGAGCCAGTTCGACATCCTGTGCAAGACCCCCCCCAAGGTGCTGGTGCGGCAGTTCGTGGAGAGATTCGAGAGGCCCAGCGGCGAGAAGATCGCCAGCTGTGCCGCCGAGCTGACCTACCTGTGCTGGATGATCACCCACAACGGCACCGCCATCAAGAGGGCCACCTTCATGAGCTACAACACCATCATCAGCAACAGCCTGAGCTTCGACATCGTGAACAAGAGCCTGCAGTTCAAGTACAAGACCCAGAAGGCCACCATCCTGGAGGCCAGCCTGAAGAAGCTGATCCCCGCCTGGGAGTTCACCATCATCCCTTACAACGGCCAGAAGCACCAGAGCGACATCACCGACATCGTGTCCAGCCTGCAGCTGCAGTTCGAGAGCAGCGAGGAGGCCGACAAGGGCAACAGCCACAGCAAGAAGATGCTGAAGGCCCTGCTGTCCGAGGGCGAGAGCATCTGGGAGATCACCGAGAAGATCCTGAACAGCTTCGAGTACACCAGCAGGTTCACCAAGACCAAGACCCTGTACCAGTTCCTGTTCCTGGCCACATTCATCAACTGCGGCAGGTTCAGCGACATCAAGAACGTGGACCCCAAGAGCTTCAAGCTGGTGCAGAACAAGTACCTGGGCGTGATCATTCAGTGCCTGGTGACCGAGACCAAGACAAGCGTGTCCAGGCACATCTACTTTTTCAGCGCCAGAGGCAGGATCGACCCCCTGGTGTACCTGGACGAGTTCCTGAGGAACAGCGAGCCCGTGCTGAAGAGAGTGAACAGGACCGGCAACAGCAGCAGCAACAAGCAGGAGTACCAGCTGCTGAAGGACAACCTGGTGCGCAGCTACAACAAGGCCCTGAAGAAGAACGCCCCCTACCCCATCTTCGCTATCAAGAACGGCCCTAAGAGCCACATCGGCAGGCACCTGATGACCAGCTTTCTGAGCATGAAGGGCCTGACCGAGCTGACAAACGTGGTGGGCAACTGGAGCGACAAGAGGGCCTCCGCCGTGGCCAGGACCACCTACACCCACCAGATCACCGCCATCCCCGACCACTACTTCGCCCTGGTGTCCAGGTACTACGCCTACGACCCCATCAGCAAGGAGATGATCGCCCTGAAGGACGAGACCAACCCCATCGAGGAGTGGCAGCACATCGAGCAGCTGAAGGGCAGCGCCGAGGGCAGCATCAGATACCCCGCCTGGAACGGCATCATCAGCCAGGAGGTGCTGGACTACCTGAGCAGCTACATCAACAGGCGGATCTAAAGGCCCTGTATGGGGTTGTGCACCGCTCCTGAATTCTGGACTTAAACACTTTAAATCCAAGGTCATAGGTTTTTTTTTCAAGCCCTAAGGGAAATGCTAGCAGTTACGGCTCGATTTGGAGGGTGAACGAGGCATAGCACGATGCACTGCATTGTTTAGATTGAAATATTTCAAAGATTAACAGGCCTCTTGCACTTATACAGTTACATTTCCATCATACCTTAATGTTATCTCTGTTTTGTGGGGTTTTTTTTACTCCTGATTTCTTTATTATCTGAAATCTTTG**

**Figure S18. Schematic and the sequence of the *Mafb*-FlpO-ssDNA. Various sequence elements are color coded. The primers used for 5’ and 3’ junction PCR and the amplicon sizes are shown above the schematic. Genotyping primer sequences are underlined.**

**FlpO 5’- R**

***Mafb* 3’- R**

**FlpO 3’- F**

***Mafb* 5’- F**

**407bp**

**407bp**

**Flanking Sequence**

**Flanking Sequence**

**FlpO**

P2A

**Left Arm**

**Right Arm**

**CTTCTCTGATGACCAGCTGGTGTCCATGTCGGTGCGTGAGCTGAACCGCCACCTGCGGGGCTTCACCAAGGACGAGGTGATCCGCCTGAAGCAGAAGCGGCGGACCCTGAAGAACCGGGGCTACGCCCAGTCGTGCAGGTATAAACGCGTCCAGCAGAAACATCACCTGGAGAACGAGAAGACGCAGCTCATTCAGCAGGTGGAGCAGCTTAAGCAGGAGGTGTCCCGGCTGGCCCGCGAGAGAGACGCCTACAAGGTCAAGTGCGAGAAACTCGCCAACTCCGGCTTCAGGGAGGCGGGCTGGTCAAGTGCGAGAAACTCGCCAACTCCGGCTTCAGGGAGGCGGGCTCCACCAGCGACAGCCCCTCCTCTCCTGAGTTCTTTCTGGGAAGCGGAGCTACTAACTTCAGCCTGCTGAAGCAGGCTGGAGACGTGGAGGAGAACCCTGGACCTGCTAGCATGGCTCCTAAGAAGAAGAGGAAGGTGATGAGCCAGTTCGACATCCTGTGCAAGACCCCCCCCAAGGTGCTGGTGCGGCAGTTCGTGGAGAGATTCGAGAGGCCCAGCGGCGAGAAGATCGCCAGCTGTGCCGCCGAGCTGACCTACCTGTGCTGGATGATCACCCACAACGGCACCGCCATCAAGAGGGCCACCTTCATGAGCTACAACACCATCATCAGCAACAGCCTGAGCTTCGACATCGTGAACAAGAGCCTGCAGTTCAAGTACAAGACCCAGAAGGCCACCATCCTGGAGGCCAGCCTGAAGAAGCTGATCCCCGCCTGGGAGTTCACCATCATCCCTTACAACGGCCAGAAGCACCAGAGCGACATCACCGACATCGTGTCCAGCCTGCAGCTGCAGTTCGAGAGCAGCGAGGAGGCCGACAAGGGCAACAGCCACAGCAAGAAGATGCTGAAGGCCCTGCTGTCCGAGGGCGAGAGCATCTGGGAGATCACCGAGAAGATCCTGAACAGCTTCGAGTACACCAGCAGGTTCACCAAGACCAAGACCCTGTACCAGTTCCTGTTCCTGGCCACATTCATCAACTGCGGCAGGTTCAGCGACATCAAGAACGTGGACCCCAAGAGCTTCAAGCTGGTGCAGAACAAGTACCTGGGCGTGATCATTCAGTGCCTGGTGACCGAGACCAAGACAAGCGTGTCCAGGCACATCTACTTTTTCAGCGCCAGAGGCAGGATCGACCCCCTGGTGTACCTGGACGAGTTCCTGAGGAACAGCGAGCCCGTGCTGAAGAGAGTGAACAGGACCGGCAACAGCAGCAGCAACAAGCAGGAGTACCAGCTGCTGAAGGACAACCTGGTGCGCAGCTACAACAAGGCCCTGAAGAAGAACGCCCCCTACCCCATCTTCGCTATCAAGAACGGCCCTAAGAGCCACATCGGCAGGCACCTGATGACCAGCTTTCTGAGCATGAAGGGCCTGACCGAGCTGACAAACGTGGTGGGCAACTGGAGCGACAAGAGGGCCTCCGCCGTGGCCAGGACCACCTACACCCACCAGATCACCGCCATCCCCGACCACTACTTCGCCCTGGTGTCCAGGTACTACGCCTACGACCCCATCAGCAAGGAGATGATCGCCCTGAAGGACGAGACCAACCCCATCGAGGAGTGGCAGCACATCGAGCAGCTGAAGGGCAGCGCCGAGGGCAGCATCAGATACCCCGCCTGGAACGGCATCATCAGCCAGGAGGTGCTGGACTACCTGAGCAGCTACATCAACAGGCGGATCTGAGTCCTGGCGGGTCCGGCCCCCGCCCTTGCCCTGGCCCAGACTCCCTATTCTGCGCCCCTAGCCCTGGACTCCCTGTCCCTGCCATGGCCCCGGCCTTGACCTGTTTGACTTGAGCTAGAGGGAGGAAGGACGCGCGGGTCGCGGGAGTCAGGCGGGAGCACGGGCGGGCAGAGAACCTTGGCTAAGAAGAGGGCAGCTCAGGGCGGCGCAGCCTCTTAGACTTGGGCAGAGTTAGAGAAACCCGGGCGGGTGCGAGGTCCGGGAGTAACTTTTCTCCAAGCTGGAAGGCCGCGAGGCTTATTCCAAGGAGTCGCCAAGGCCGTCTGGACACTTCTGGCTCTGAGAACTTTGTGAGTGCCAGGGCCGCCGCTTTGCAGCC**

**Figure S19. Schematic and sequence of the *Otoa*-rtTA ssDNA. Various sequence elements are color coded. The primers used for 5’ and 3’ junction PCR and the amplicon sizes are shown above the schematic. Genotyping primer sequences are underlined.**

**1065bp**

**rtTA 5’- R**

***Otoa* 3’- R**

**rtTA 3’- F**

**465bp**

***Otoa* 5’- F**

**Flanking Sequence**

**Right Arm**

PolyA

**Flanking Sequence**

rtTA

**Left Arm**

AAGATGGTGCAGCAAGCACTTTGAAACACTCTTCTCCTCTGGGTGCTGTGGACCAGGGGTGGTGGTGAGCACAGAAACGGGGCGCTGAGGTCACTGAATGATGGACATGTCTTAGACACACCCTGGAAT**CATCACACTACTTAAGATGGTTTTGAGG**GGTTTAGT**GGCTGCATGTCCCTCTGCTCCTGAATATTCTTCAATCTACTTTTAAAAGAAAATGAAACCTGAGTCACCTGTGTGCTTGACTTAACTGCAGGATGTCTAGACTGGACAAGAGCAAAGTCATAAACTCTGCTCTGGAATTACTCAATGGAGTCGGTATCGAAGGCCTGACGACAAGGAAACTCGCTCAAAAGCTGGGAGTTGAGCAGCCTACCCTGTACTGGCACGTGAAGAACAAGCGGGCCCTGCTCGATGCCCTGCCAATCGAGATGCTGGACAGGCATCATACCCACTCCTGCCCCCTGGAAGGCGAGTCATGGCAAGACTTTCTGCGGAACAACGCCAAGTCATACCGCTGTGCTCTCCTCTCACATCGCGACGGGGCTAAAGTGCATCTCGGCACCCGCCCAACAGAGAAACAGTACGAAACCCTGGAAAATCAGCTCGCGTTCCTGTGTCAGCAAGGCTTCTCCCTGGAGAACGCACTGTACGCTCTGTCCGCCGTGGGCCACTTTACACTGGGCTGCGTATTGGAGGAACAGGAGCATCAAGTAGCAAAAGAGGAAAGAGAGACACCTACCACCGATTCTATGCCCCCACTTCTGAAACAAGCAATTGAGCTGTTCGACCGGCAGGGAGCCGAACCTGCCTTCCTTTTCGGCCTGGAACTAATCATATGTGGCCTGGAGAAACAGCTAAAGTGCGAAAGCGGCGGGCCGACCGACGCCCTTGACGATTTTGACTTAGACATGCTCCCAGCCGATGCCCTTGACGACTTTGACCTTGATATGCTGCCTGCTGACGCTCTTGACGATTTTGACCTTGACATGCTCCCCGGGTAACTAAGTAAGGATCCAGACATGATAAGATACATTGATGAGTTTGGACAAACCACAACTAGAATGCAGTGAAAAAAATGCTTTATTTGTGAAATTTGTGATGCTATTGCTTTATTTGTAACCATTATAAGCTGCAATAAACAAGTTAACAACAACAATTGCATTCATTTTATGTTTCAGGTTCAGGGGGAGGTGTGGGAGGTTTTTTAAAGCAAGTAAAACCTCTACAAATGTGGTATGGCTGATTATGATCCTGCAAGCCTCGTCGTCCTGGCCGGACCACGCTATCTGTGCAAGGTCCCCGGCCCCGGACGCGCGCTCCATGAGCAGAGCGCCCGCCGCCGAGGCGAAGACTCGGGCGGCGCCCTGCCCGTCCCACCAGGTCAACAGGCGGTAACCGGCCTCTTCATCGGGAATGCGCGCGACCTTCAGCATCGCCGGCATGTCCCCCTGGCGGACGGGAAGTATCCAGCTCGACCACTCAGGGACCTAGGACGTGCTCCCTTCTCCTGGTCCTCCTTCTGAGCCATGGAGGTGCCTATCAGAGAGAGCCAAGCCCCAGGCAGGGTAAGTCTTGA**GGAGAGAGAGAGAGGGAGAGAGAGAGAGAGAGAGAGAGAGAGAGAGAGAGAGAGATTGATTTCACAGCTCCTATATATTTTAGGGGATGTAGCTGGATATGTCCGTTTGGGGTTATGATATATTCCAGTGAGGGGGTAGTAAGCATTTGCCTATCTGTGGGTATTCATAGGGCTTTTGGGAGTATTATACATGGTTTATTTTATTATAATGTGTATTATTTATCCAAATAAATGTATTATGCCACTTTCATGCATGCACACAATATGCTTCAGTGGAATTCACCCCCAACACCCTCTCTTCCCC**CATTCCTCACAAGTCCAGTAGATCC**CACACATGAGGGAAGCTTGCAGTGCCTGTCTTCCTGAGTCTGACCCATGTTGATTAACGTGATGATCTCCGGTTCCAACATTTCTCCTGCAAATGACAAAACTTCATTCTTCTTTATGGTCAAATAATACTCTACCTGCT

**Figure S20.** **Schematic and sequence of the *Mmp9*-T2A-mCitrine-ssDNA. Various sequence elements are color coded. The primers used for 5’ and 3’ junction PCR and the amplicon sizes are shown above the schematic. Genotyping primer sequences are underlined.**

**mCitrine 3’- F**

**mCitrine 5’- R**

***Mmp9* 3’- R**

**561bp**

***Mmp9* 5’- F**

**263bp**

**Flanking Sequence**

**Right Arm**

mCitrine

**Left Arm**

**Flanking Sequence**

T2A

**GCAAACGTGGTGTTGGAGAGGTAGCTGAGAGCTCTACCTCAGCAGCAGGCAGCAAGAAGAGAGAGACACTGGGCTTGGCGTGAACATCTGAAATCTCAAAGCCCACCCTAGTGACACAGGTCCTCCAATAAGGCCACACCCCCTCCCACCTCATAAGTGCCACTCCCTATGGGCCTATAGGGTCCATTTTCATTCAAACCACCACAGGTGCTTGTAAAAATCTTGTTCCTTTTGTCAATGATGAGGGAGCAGAGGACTCAGCTTTCTTGTGTGTTCTTGTTTCCCGTAGACAAAGCCTATTTCTGCCATGGCAAATTCTTCTGGCGTGTGAGTTTCCAAAATGAGGTGAACAAGGTGGACCATGAGGTGAACCAGGTGGACGACGTGGGCTACGTGACCTACGACCTCCTGCAGTGCCCTGGCAGTGGAGAGGGCAGAGGAAGTCTGCTAACATGCGGTGACGTCGAGGAGAATCCTGGCCCAATGGTGAGCAAGGGCGAGGAGCTGTTCACCGGGGTGGTGCCCATCCTGGTCGAGCTGGACGGCGACGTAAACGGCCACAAGTTCAGCGTGTCCGGCGAGGGCGAGGGCGATGCCACCTACGGCAAGCTGACCCTGAAGTTCATCTGCACCACCGGCAAGCTGCCCGTGCCCTGGCCCACCCTCGTGACCACCTTCGGCTACGGCCTGATGTGCTTCGCCCGCTACCCCGACCACATGAAGCAGCACGACTTCTTCAAGTCCGCCATGCCCGAAGGCTACGTCCAGGAGCGCACCATCTTCTTCAAGGACGACGGCAACTACAAGACCCGCGCCGAGGTGAAGTTCGAGGGCGACACCCTGGTGAACCGCATCGAGCTGAAGGGCATCGACTTCAAGGAGGACGGCAACATCCTGGGGCACAAGCTGGAGTACAACTACAACAGCCACAACGTCTATATCATGGCCGACAAGCAGAAGAACGGCATCAAGGTGAACTTCAAGATCCGCCACAACATCGAGGACGGCAGCGTGCAGCTCGCCGACCACTACCAGCAGAACACCCCCATCGGCGACGGCCCCGTGCTGCTGCCCGACAACCACTACCTGAGCTACCAGTCCGCCCTGAGCAAAGACCCCAACGAGAAGCGCGATCACATGGTCCTGCTGGAGTTCGTGACCGCCGCCGGGATCACTCTCGGCATGGACGAGCTGTACAAGTGAACTAGGGCTCCTTCTTTGCTTCAACCGTGCAGTGCAAGTCTCTAGAGACCACCACCACCACCACCACACACAAACCCCATCCGAGGGAAAGGTGCTAGCTGGCCAGGTACAGACTGGTGATCTCTTCTAGAGACTGGGAAGGAGTGGAGGCAGGCAGGGCTCTCTCTGCCCACCGTCCTTTCTTGTTGGACTGTTTCTAATAAACACGGATCCCCAACCTTTTCCAGCTACTTTAGTCAATCAGCTTATCTGTAGTTGCAGATGCATCCGAGCAAGAAGACAACTTTGTAGGGTGGATTCTGACCTTTTATTTTTGTGTGGCGTCTGAGAAT**

**Figure S21.** **Schematic and sequence of the *Mmp13*-T2A-mCherry-ssDNA. Various sequence elements are color coded. The primers used for 5’ and 3’ junction PCR and the amplicon sizes are shown above the schematic. Genotyping primer sequences are underlined.**

**mCherry 3’- F**

**mCherry 5’- R**

***Mmp13* 3’- R**

**556bp**

***Mmp13* 5’- F**

**525bp**

mCherry

**Flanking Sequence**

**Right Arm**

**Flanking Sequence**

**Left Arm**

T2A

**AGGTGAATCTCAGGCACTGCAGTAACCAATTGTAATTGATAATTCCCATTGTAATTAAAGTAAAAATAACTGTATAGTTATGATAATTATGAATCTTTATAAATTACTCTTCACTCCAGCCCTAATATGAATTTTGACATAACCCTCTGCCTCTTGACACTTTTACATGTAACTTAAGGTCATCCTGAGGTTCAACATCTGTTGTTCACACACTTGGTAGTAGCTAAGTAAAATTCTCATTGATTCTAACAGGATATTTTGTTTGCTATCTAAGTAACCAATTGAATATTTAGCAAAGGACTATCACAGATTAAATCTCCAATCTTAGCTGCAGTAAATCCTAATATTTTTAAAGTCTGAGATTTTTTCTTTTGTTCTCTTTCTTCCTAGGCTATATCTACTTTTTCAATGGGCCCATACAGTTTGAATACAGTATCTGGAGTAATCGCATTGTGAGAGTCATGCCAACAAATTCCATAT**TGTGGTGT**GGCAGTGGAGAGGGCAGAGGAAGTCTGCTAACATGCGGTGACGTCGAGGAGAATCCTGGCCCAATGGTGAGCAAGGGCGAGGAGGATAACATGGCCATCATCAAGGAGTTCATGCGCTTCAAGGTGCACATGGAGGGCTCCGTGAACGGCCACGAGTTCGAGATCGAGGGCGAGGGCGAGGGCCGCCCCTACGAGGGCACCCAGACCGCCAAGCTGAAGGTGACCAAGGGTGGCCCCCTGCCCTTCGCCTGGGACATCCTGTCCCCTCAGTTCATGTACGGCTCCAAGGCCTACGTGAAGCACCCCGCCGACATCCCCGACTACTTGAAGCTGTCCTTCCCCGAGGGCTTCAAGTGGGAGCGCGTGATGAACTTCGAGGACGGCGGCGTGGTGACCGTGACCCAGGACTCCTCCCTGCAGGACGGCGAGTTCATCTACAAGGTGAAGCTGCGCGGCACCAACTTCCCCTCCGACGGCCCCGTAATGCAGAAGAAGACCATGGGCTGGGAGGCCTCCTCCGAGCGGATGTACCCCGAGGACGGCGCCCTGAAGGGCGAGATCAAGCAGAGGCTGAAGCTGAAGGACGGCGGCCACTACGACGCTGAGGTCAAGACCACCTACAAGGCCAAGAAGCCCGTGCAGCTGCCCGGCGCCTACAACGTCAACATCAAGTTGGACATCACCTCCCACAACGAGGACTACACCATCGTGGAACAGTACGAACGCGCCGAGGGCCGCCACTCCACCGGCGGCATGGACGAGCTGTACAAGTAAGCATCTTTAAAAGTTGTTATTTATCTCCCAGAGAGTATTTGGAATACTTTCAGATGTATGGGGTGGGGGTGGGGTGGAGATATCAGGGGAGAGCTTAGTTCTGTGAACGAGCTTCAGTAAGTTATCTTTGAGCATACAGTATCTATATGACTATGCGTGGCTGGAACCACATGGAAGAATTTTAAAGTAATGCAATTGAGAACCCCAAGGATCACCTGATTCTTGCGTGCTATGAAGAAACAAGATTGATAATAACCCACAGCAAACATGGGGTCCATCTGCTTTTGAGAGCATGCATAATTATTAATATATTTATTTTAAAAAGCCTAACAGACATAAAATAAATCATATTTATATAACTGAATTGTCTTTACAAAAAAGTATAAACTTAGA**

**Figure S22: Reproducibility of *Easi*-CRISPR.** Experiments to generate *Fgf8*^P2A-FlpO^ and *Otoa^rtTA^* mice were repeated. (**A**) Genotyping of animals generated from *Fgf8* microinjections showing 2 out of 6 (animals #5 and #7) contained the knock-in cassette (33% efficiency). Animal #8 contained the cassette but it had a deletion in the 5’ end. The genotyping schematics and assay details are as described in **Figure 3.** **(B)** Genotyping of animals generated from *Otoa* microinjections showing 3 out of 8 (animals #3, #5 and #8) contained the knock-in cassette (37.5% efficiency). The genotyping schematics and assay details are as described in Additional file 1: **Figure S13**. **(C, D)** Microinjection details.

| **Table S1. Primer sequences used in the study** |
| --- |
| **Locus** **Primer sequence** |
| Pitx1 5’F CACAGGAACGGTTTTGACTTC |
| Pitx1 5’R TTGTCTGCAGCAGGTAGGAA |
| Pitx1 3’F ACATGAGCATGAGAGAGGAGA |
| Pitx1 3’R TACAGTGGGAAAGGGCATTAG |
| Ambra1 5’F AAGCACTGGATGAATGTGTG |
| Ambra1 5’R ATGTGTGGATGCCAAGAGAG |
| Ambra1 3’F TTTCACCCCACCATCTCAG |
| Ambra1 3’R GCAAACATAGTGAAAGCAACC |
| Ambra1 5’ Lox F ATTGCTTTGCTTGACCAGTAATAACTTCGTATAGC |
| Ambra1 3’ Lox R GCAATGTCTACCAGTCCTAACAATA |
| Col12a1 5’ F GCAGTATGAAGTCATGTGCGG |
| Col12a1 5’ R ACCTTCTGCCTCAATGGAAGAC |
| Col12a1 3’ F ACTCCTGTCTTCCATTGAGGC |
| Col12a1 3’ R AAGGTCACAGTCCTGACCCA |
| Ubr5 5’ F GGATTAAAGGTGTGCGCTGCC |
| Ubr5 5’ R GAGACTTCTCACTCACCAGG |
| Ubr5 3’ F GCTCCTCCAGTTCAAACGGT |
| Ubr5 3’ R GACCCAAACAATGACTCAGAAC |
| Ppp2r2a 5’ F CCTGTTCTACAAACTTGCCCTCTG |
| Ppp2r2a 5’ R CTCCTGTGGCTAGTAATTCTCC |
| Ppp2r2a 3’ F GTGCATAGATGGAGAATGCTGA |
| Ppp2r2a 3’ R CAGAGCAGCTAAGCCTTGAG |
| FGF8 5’ F TCACAGAGATCGTGCTGGAGAAC |
| FGF8 3’ R CCAACAGCAAACAATATGC |
| Pitx1 5’Lox F CAAGAACAACATTCCAGGCATAACTTCGTATAGC |
| Pitx1 3’Lox R AGGGGCTTGCAGTAGCTCATAACTTCGTATAAT |
| Slc26 5’ F AATCCTGCCTTGAAAGAGCTTCTG |
| Slc26 3’ R CCTCCAAATCGAGCCGTAACTG |
| Mafb 5’ F CAGAAACATCACCTGGAGAACGAG |
| Mafb 3’ R GGTTTCTCTAACTCTGCCCAAGTC |
| FlpO 5’ R CCTCTCGAATCTCTCCACGAACTG |
| FlpO Int F TGAGCTTCGACATCGTGAAC (sequencing primer) |
| FlpO Int R TCAGCATCTTCTTGCTGTGG (sequencing primer) |
| FlpO Int F1 TCCTGAACAGCTTCGAGTACACC (sequencing primer) |
| FlpO 5’ R2 AAAGTAGATGTGCCTGGACACG (sequencing primer) |
| FlpO Int F2 CAGCAACAAGCAGGAGTACCAG (sequencing primer) |
| FlpO 3’ F GAGATGATCGCCCTGAAGGAC |
| Otoa 5’ F CATCACACTACTTAAGATGGTTTTGAGG |
| rtta 5’ R GGGTTTCGTACTGTTTCTCTGTTGG |
| rtta 3’ F GCCTGGAGAAACAGCTAAAGTGCGAAAGC |
| Otoa 3’ R GGATCTACTGGACTTGTGAGGAATG |
| rtta Int F1 CTCCCTGGAGAACGCACTGTAC (sequencing primer) |
| rtta Int F2 AAGAGAGACACCTACCACCGATTC (sequencing primer) |
| rtta Int R1 CTGCATTCTAGTTGTGGTTTGTCC (sequencing primer) |
| Mmp9 5’F TGGCGTGAACATCTGAAATC |
| mCitrine 5’R GGTGGTGCAGATGAACTTCAG |
| mCitrine 3’F ATGGTCCTGCTGGAGTTCGT |
| Mmp9 3’R GAAACAGTCCAACAAGAAAGGAC |
| Mmp13 5’F ACTCTTCACTCCAGCCCTAATATG |
| mCherry 5’R TCGCCCTCGATCTCGAAC |
| mCherry 3’F ATGCAGAAGAAGACCATGGGC |
| Mmp13 3’R CATAGCACGCAAGAATCAGG |
| Syt1 5’ F GAGATTCGTACAAGATGGCAGAGC |
| Syt1 5 R GATCCTTCATGGTCTTCCCTAAGTC |
| Syt1 3’F GGAGGGAAGAACGCCATTAAC |
| Syt1 3’ R GCATTGCCAAGTGGATGAAG |
| Syt9 5’F GCATCAATGGGAGGAGAGGTCA |
| Syt9 5’R AGGGTCTTCCTGTGAACTTTAG |
| Syt9 3’ F GAAGATCCACAAAGCCGTCAAT |
| Syt9 3’R GATGCCATGACCCAGTTAATAC |
